# Expanding the Enzyme Repertoire for Sugar Nucleotide Epimerization: the CDP-Tyvelose 2-Epimerase from Thermodesulfatator atlanticus for Glucose/Mannose Interconversion

**DOI:** 10.1128/AEM.02131-20

**Published:** 2021-01-29

**Authors:** Christian Rapp, Stevie van Overtveldt, Koen Beerens, Hansjörg Weber, Tom Desmet, Bernd Nidetzky

**Affiliations:** a Institute of Biotechnology and Biochemical Engineering, Graz University of Technology, NAWI Graz, Graz, Austria; b Centre for Synthetic Biology, Department of Biotechnology, Ghent University, Ghent, Belgium; c Institute of Organic Chemistry, Graz University of Technology, NAWI Graz, Graz, Austria; d Austrian Centre of Industrial Biotechnology (acib), Graz, Austria; Shanghai Jiao Tong University

**Keywords:** CDP-glucose, carbohydrate synthesis, catalytic mechanism, epimerase, multistep enzyme catalysis, short-chain dehydrogenase/reductase (SDR), specificity, sugar nucleotide

## Abstract

Epimerization of sugar nucleotides is central to the structural diversification of monosaccharide building blocks for cellular biosynthesis. Epimerase applicability to carbohydrate synthesis can be limited, however, by the high degree of substrate specificity exhibited by most sugar nucleotide epimerases. Here, we discovered a promiscuous type of CDP-tyvelose 2-epimerase (TyvE)-like enzyme that promotes C-2 epimerization in all nucleotide (CDP, UDP, GDP, ADP, and TDP)-activated forms of d-glucose. This new epimerase, originating from Thermodesulfatator atlanticus, is a functional homodimer that contains one tightly bound NAD^+^/subunit and shows optimum activity at 70°C and pH 9.5. The enzyme exhibits a *k*_cat_ with CDP-d-glucose of ∼1.0 min^−1^ (pH 7.5 and 60°C). To characterize the epimerase kinetically and probe its substrate specificity, we developed chemoenzymatic synthesis for CDP-d-mannose, CDP-6-deoxy-d-glucose, CDP-3-deoxy-d-glucose, and CDP-6-deoxy-d-*xylo*-hexopyranos-4-ulose. Attempts to obtain CDP-d-paratose and CDP-d-tyvelose were not successful. Using high-resolution carbohydrate analytics and *in situ* nuclear magnetic resonance (NMR) to monitor the enzymatic conversions (60°C and pH 7.5), we show that the CDP-d-mannose/CDP-d-glucose ratio at equilibrium is 0.67 (±0.1), determined from the kinetic Haldane relationship and directly from the reaction. We further show that deoxygenation at sugar C-6 enhances the enzyme activity 5-fold compared to CDP-d-glucose, whereas deoxygenation at C-3 renders the substrate inactive. Phylogenetic analysis places the T. atlanticus epimerase into a distinct subgroup within the sugar nucleotide epimerase family of SDRs (short-chain dehydrogenases/reductases), for which the current study now provides functional context. Collectively, our results expand an emerging toolbox of epimerase-catalyzed reactions for sugar nucleotide synthesis.

**IMPORTANCE** Epimerases of the sugar nucleotide-modifying class of enzymes have attracted considerable interest in carbohydrate (bio)chemistry for the mechanistic challenges and the opportunities for synthesis involved in the reactions catalyzed. The discovery of new epimerases with an expanded scope of sugar nucleotide substrates used is important to promote mechanistic inquiry and can facilitate the development of new enzyme applications. Here, a CDP-tyvelose 2-epimerase-like enzyme from Thermodesulfatator atlanticus is shown to catalyze sugar C-2 epimerization in CDP-glucose and other nucleotide-activated forms of d-glucose. The reactions are new to nature in the context of enzymatic sugar nucleotide modification. The current study explores the substrate scope of the discovered C-2 epimerase and, based on modeling, suggests structure-function relationships that may be important for specificity and catalysis.

## Abstract (precis)

Epimerases of the sugar nucleotide-modifying class of enzymes have attracted considerable interest in carbohydrate (bio)chemistry for the mechanistic challenges and the opportunities for synthesis involved in the reactions catalyzed. The discovery of new epimerases with an expanded scope of sugar nucleotide substrates used is important to promote mechanistic inquiry and can facilitate the development of new enzyme applications.

## INTRODUCTION

Epimerization (configuration change at a single stereocenter of diastereomers) is an attractive transformation for carbohydrate synthesis since it enables the direct interconversion of monosaccharide structures. Such epimerization is promising in particular when valuable products can be obtained from naturally abundant sugar substrates (e.g., d-glucose) (1–6). Sugar epimerization has been long known to carbohydrate chemistry (7), but the underlying reactions are usually complex. Despite notable progress recently (8, 9), purely chemical epimerization remains challenging to establish for the structurally well-defined synthesis of carbohydrates. Epimerization that exploits the high regioselectivity of enzymes for synthetic precision would be highly desirable (10, 11).

Enzymatic epimerization reactions can be distinguished according to whether they require an activated form of the sugar, typically a sugar nucleotide, as the substrate (12, 13). Transformations of nonactivated substrates can be efficient due to their overall simplicity. The conversion of d-fructose into d-allulose (d-psicose) by d-tagatose 3-epimerase is representative and has industrial importance (14, 15). However, the type of catalysis used by these epimerases, effectively a combination of concerted general acid/base catalysis, limits the scope of transformations available to plain monosaccharides (13, 16). Epimerases acting on sugar nucleotide substrates offer a richer portfolio of catalytic chemistry, including oxidation/reduction and elimination/readdition (17–22). They can thus expand the range of substrate transformations accessible for epimerization (10). The epimerized sugar in the nucleotide-activated form can be further utilized by glycosyltransferases in oligosaccharide and glycosylated natural product synthesis (23–25).

Sugar nucleotide-dependent epimerases hence complement the synthetic repertoire and the applied perspective of enzymatic epimerization. However, limitations in their applicability to carbohydrate synthesis can arise from the high substrate specificity that many of these enzymes exhibit (19, 21, 26). Novel epimerases more promiscuous in their acceptance of sugar nucleotide substrates are hence of high interest. Here, we addressed epimerization at sugar C-2 in unmodified d-glucose nucleotide substrates to establish the corresponding d-mannose configuration. This apparently simple transformation is not known to nature at the level of the sugar nucleotide. C-2 epimerization of the d-glucose configuration is found in nonactivated substrates (cellobiose [27, 28] and *N*-acetylglucosamine [29]) and in the activated UDP-*N*-acetylglucosamine, in which the C-2 is modified (30).

**FIG 1:**
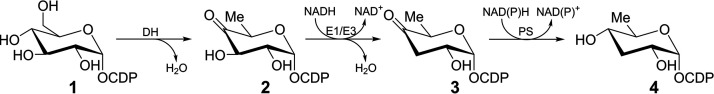
Biosynthesis of CDP-d-paratose (33–35). Compound 1, CDP-d-glucose; compound 2, CDP-6-deoxy-d-*xylo*-hexopyranos-4-ulose; compound 3, CDP-3,6-dideoxy-d-*xylo*-hexopyranos-4-ulose; compound 4, CDP-d-paratose; DH, CDP-d-glucose 4,6-dehydratase (EC 4.2.1.45); E1/E3, CDP-4-keto-6-deoxy-d-glucose-3-dehydrogenase system (EC 1.17.1.1); PS, CDP-d-paratose synthase (EC 1.1.1.342).

An interesting candidate enzyme for our purpose was CDP-d-tyvelose 2-epimerase (other name, CDP-d-paratose 2-epimerase) (EC 5.1.3.10) (19), referred to here as TyvE. d-Paratose (3,6-dideoxy-d-ribo-hexopyranose) is a rare dideoxy sugar discovered from the O-antigen glycans of human-pathogenic bacteria such as *Salmonella* sp. and *Yersinia* sp. (31, 32). The integration of d-paratose into O-antigen glycans requires the CDP-activated form. The biosynthesis of CDP-d-paratose (CDP-Par) starts from CDP-d-glucose (CDP-Glc) and proceeds via 4,6-dehydration, 3-deoxygenation, and 4-keto group reduction (33–35) (Fig. 1).

Mechanistically, the TyvE reaction involves a transient C-2 keto intermediate owing to an overall epimerization that is comprised of two half-reactions, oxidation and reduction (19). Tightly bound NAD^+^ is the enzyme’s cofactor to mediate hydride transfer from and to the sugar C-2. From its sequence and three-dimensional structure (36), TyvE is classified as a member of the family of sugar nucleotide epimerases (12) within the short-chain dehydrogenase/reductase (SDR) protein superfamily (37–39). Although d-paratose is substantially modified from d-glucose, it retains the reactive C-2 hydroxy group. CDP-Glc is shown to be unreactive with TyvE from Yersinia pseudotuberculosis (19).

The point of departure for this study was an extensive database search for putative TyvE-like epimerases. Sequence comparisons of relevant hits revealed a new subgroup of SDR epimerases, about 65% identical in sequence to functionally characterized TyvE from Y. pseudotuberculosis (ypTyvE) and Salmonella enterica serovar Typhi (stTyvE) (19, 36, 40, 41). We envisioned that enzymes from this subgroup would retain the basic C-2 epimerase activity but might exhibit altered substrate specificity compared to TyvE. We thus identified a promiscuous TyvE-like epimerase from Thermodesulfatator atlanticus (a thermophilic marine bacterium) (42) that promotes C-2 epimerization in most of the nucleotide (CDP, UDP, GDP, ADP, and TDP)-activated forms of d-glucose. Here, we report the discovery and biochemical characterization of this new enzyme, referred to as *Ta*CPa2E, and present a detailed kinetic analysis of the reaction(s) catalyzed by it.

## RESULTS AND DISCUSSION

### Discovery of a CDP-Glc-active C-2 epimerase from Thermodesulfatator atlanticus.

**FIG 2:**
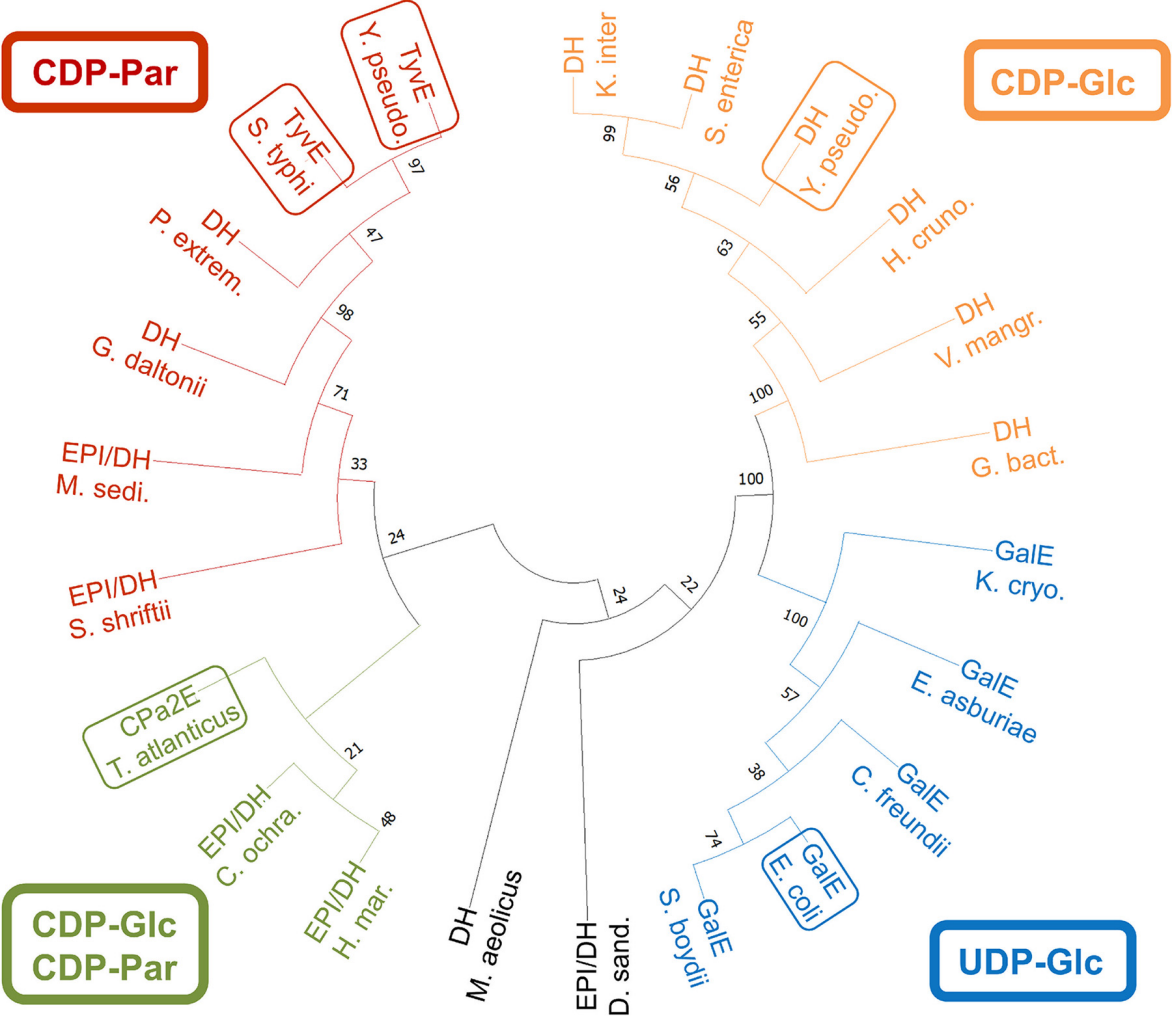
Functional clustering and phylogeny of SDR-type sugar nucleotide C-2/C-4 epimerases and dehydratases. The phylogenetic tree shows selected relevant hits from a sequence database search using ypTyvE as the template. Putative and characterized (framed) TyvEs, GalEs, and CDP-d-glucose 4,6-dehydratases are shown and grouped according to substrate specificity (confirmed biochemically for at least one member in each group). The putative CDP-sugar C-2 epimerase from T. atlanticus (green frame) is representative of a number of similar proteins from extremophile marine bacteria. CPa2E T. atlanticus, epimerase/dehydratase from Thermodesulfatator atlanticus (NCBI accession number WP_022854415.1); EPI/DH H. mar., epimerase/dehydratase from Halobacteriovorax marinus (NCBI accession number WP_096908434.1); EPI/DH C. ochra., epimerase/dehydratase from Capnocytophaga ochracea (NCBI accession number WP_015781970.1); TyvE S. typhi, epimerase from *Salmonella* Typhi (NCBI accession number WP_000770936); TyvE Y. pseudo., epimerase from Yersinia pseudotuberculosis (NCBI accession number AKA20962.1); DH P. extrem., CDP-d-glucose 4,6-dehydratase from Pseudomonas extremaustralis (NCBI accession number WP_010566515.1); DH G. daltonii, CDP-d-glucose 4,6-dehydratase from Geobacter daltonii (NCBI accession number WP_012647211.1); EPI/DH S. shriftii, epimerase/dehydratase from Selenihalanaerobacter shriftii (NCBI accession number WP_078811136.1); EPI/DH M. sedi., epimerase/dehydratase from Mangrovitalea sediminis (NCBI accession number WP_097460147.1); DH Y. pseudo., CDP-d-glucose 4,6-dehydratase from Yersinia pseudotuberculosis (NCBI accession number WP_072080415.1); DH S. enterica, CDP-d-glucose 4,6-dehydratase from Salmonella enterica (NCBI accession number EAA4947084.1); DH K. inter, CDP-d-glucose 4,6-dehydratase from Kluyvera intermedia (NCBI accession number WP_062778321.1); DH H. cruno., CDP-d-glucose 4,6-dehydratase from Hydrogenovibrio crunogenus (NCBI accession number WP_011371110.1); DH G. bact., CDP-d-glucose 4,6-dehydratase from *Gallionellaceae* bacterium (NCBI accession number TAJ82294.1); DH V. mangr., CDP-d-glucose 4,6-dehydratase from Vibrio mangrovi (NCBI accession number WP_087481551.1); GalE E. coli, UDP-d-glucose 4-epimerase from Escherichia coli (NCBI accession number 1LRJ_A); GalE E. asburiae, UDP-d-glucose 4-epimerase from Enterobacter asburiae (NCBI accession number WP_057060194.1); GalE K. cryo., UDP-d-glucose 4-epimerase from Kluyvera cryocrescens (NCBI accession number WP_061282664.1); GalE C. freundii, UDP-d-glucose 4-epimerase from Citrobacter freundii (NCBI accession number WP_086503853.1); GalE S. boydii, UDP-d-glucose 4-epimerase from Shigella boydii (NCBI accession number WP_073817003.1); EPI/DH D. sand., epimerase/dehydratase from Dethiosulfatarculus sandiegensis (NCBI accession number WP_044352701.1); DH M. aeolicus, CDP-d-glucose 4,6-dehydratase from Methanococcus aeolicus (NCBI accession number WP_011973107.1).

A database search using the TyvE from Y. pseudotuberculosis as the query and subsequent phylogenetic analysis of relevant hits (Fig. 2) revealed a new subgroup of TyvE-like proteins, annotated as epimerases or 4,6-dehydratases. With a few exceptions from mesophilic organisms, the proteins are mostly from extremophilic marine sulfate- or iron-reducing bacteria and exhibit about 60 to 70% sequence identity to ypTyvE. Using motif-based sequence analysis, we found that residues of the immediate catalytic center (Thr124, Tyr164, and Lys168) as well as residues for binding of the nicotinamide cofactor (Gly7, Gly10, and Gly13) and cytidine 5′-diphosphate (Trp207, Trp210, and Phe211) are conserved in stTyvE and ypTyvE (for the full sequence alignment, see Fig. S1 in the supplemental material). Therefore, TyvE-like proteins are likely to retain basic characteristics of epimerase function, including activity with a CDP-activated sugar. The protein from Thermodesulfatator atlanticus (42) proved to be a suitable candidate for further study as it was found to be stable under the assay conditions applied (pH 7.5 at 60°C) and over a storage time of ≥52 weeks at −20°C.

The codon-optimized gene for the protein harboring a C-terminal His tag was cloned into a pET21a vector and expressed in Escherichia coli BL21(DE3). The purified protein was obtained at a yield of ∼45 mg/liter culture (Fig. S2). Like other SDR-type epimerases, the protein is a homodimer, as shown by gel filtration (Fig. S3 and S4), and contains a tightly bound nicotinamide cofactor, as indicated by the enzyme’s absorbance spectrum (Fig. S5). High-performance liquid chromatography (HPLC) analysis (21) revealed NAD (not NADP) to be the cofactor, being present predominantly in the oxidized form (Fig. S6) (NAD^+^, 98.1% ± 0.4%; NADH, 1.9% ± 0.4%). The enzyme was found to be stable under the assay conditions applied (pH 7.5 at 60°C) and over a storage time of ≥52 weeks at −20°C.

**FIG 3:**
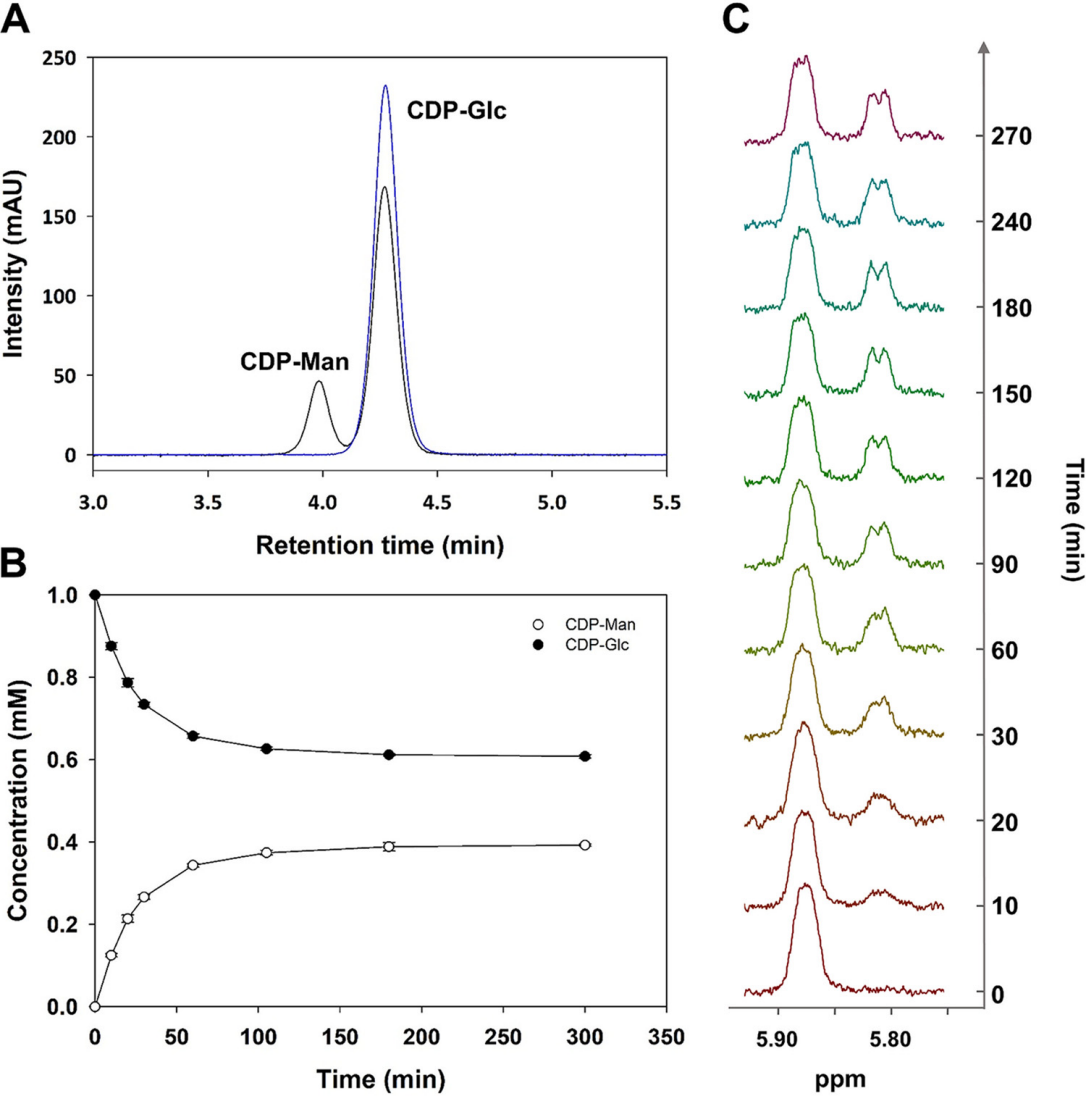
Analysis of the reaction of *Ta*CPa2E with CDP-Glc. (A) HPLC chromatogram of the reaction mixture of *Ta*CPa2E with CDP-Glc (1 mM) after 20 min (black), showing an additional peak assigned as CDP-Man. The control without the enzyme (blue) is also shown. The intensity signal is given in mAU (milliampere units). (B) Time course for the conversion of CDP-Glc (1 mM) to CDP-Man using *Ta*CPa2E (1 mg/ml; 25.4 μM) at 60°C and pH 7.5 analyzed by HPLC (λ = 271 nm). (C) *In situ* ^1^H NMR snapshot from the anomeric region with CDP-Glc (0 to 270 min). Certain time points of the signals from the anomeric protons of CDP-Glc (5.87 ppm) and CDP-Man (5.78 ppm) are shown. Conditions were CDP-Glc (4 mM) and 15.2 μM *Ta*CPa2E (0.6 mg/ml) at 60°C with 50 mM potassium phosphate buffer (pD 7.5).

**FIG 4:**
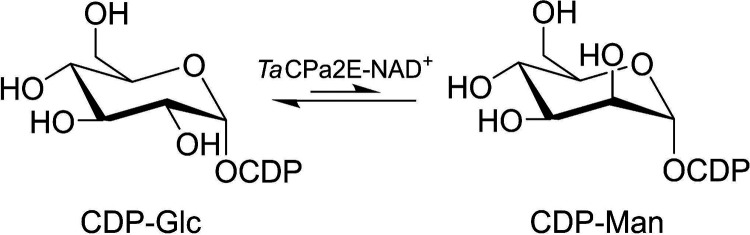
Interconversion of CDP-Glc and CDP-Man by *Ta*CPa2E.

To assess the protein for its potential C-2 epimerase activity with CDP-Glc, we needed to develop synthesis procedures for both the substrate and the expected product, CDP-d-mannose (CDP-Man). The approaches used for synthesis are described below. Offering CDP-Glc (1 mM) to the purified protein (25.4 μM), we showed by HPLC that the substrate was converted into a single detectable product that was confirmed to be CDP-Man (Fig. 3A). Following the conversion of CDP-Glc (pH 7.5 at 60°C) over time (Fig. 3B), the reaction equilibrated at an equilibrium constant (*K*_eq_) of [CDP-Man]/[CDP-Glc] of 0.67 ± 0.1 (*n *= 6). The addition of NAD^+^ (up to 1,000 μM) did not affect the reaction rate or the *K*_eq_ (Fig. S7). A proton NMR study with a D_2_O solvent was conducted to record epimerization *in situ* (Fig. 3C). Anomeric proton signals could be tracked conveniently, and an equilibrium ratio of CDP-Man/CDP-Glc of ∼0.7 was found, consistent with results obtained with H_2_O. NMR data additionally corroborate that CDP-Man is the sole product of the enzymatic reaction. We confirmed the identity of the product and thus demonstrate the new enzymatic reactivity as epimerization at the C-2 of CDP-Glc (Fig. 4).

**FIG 5:**
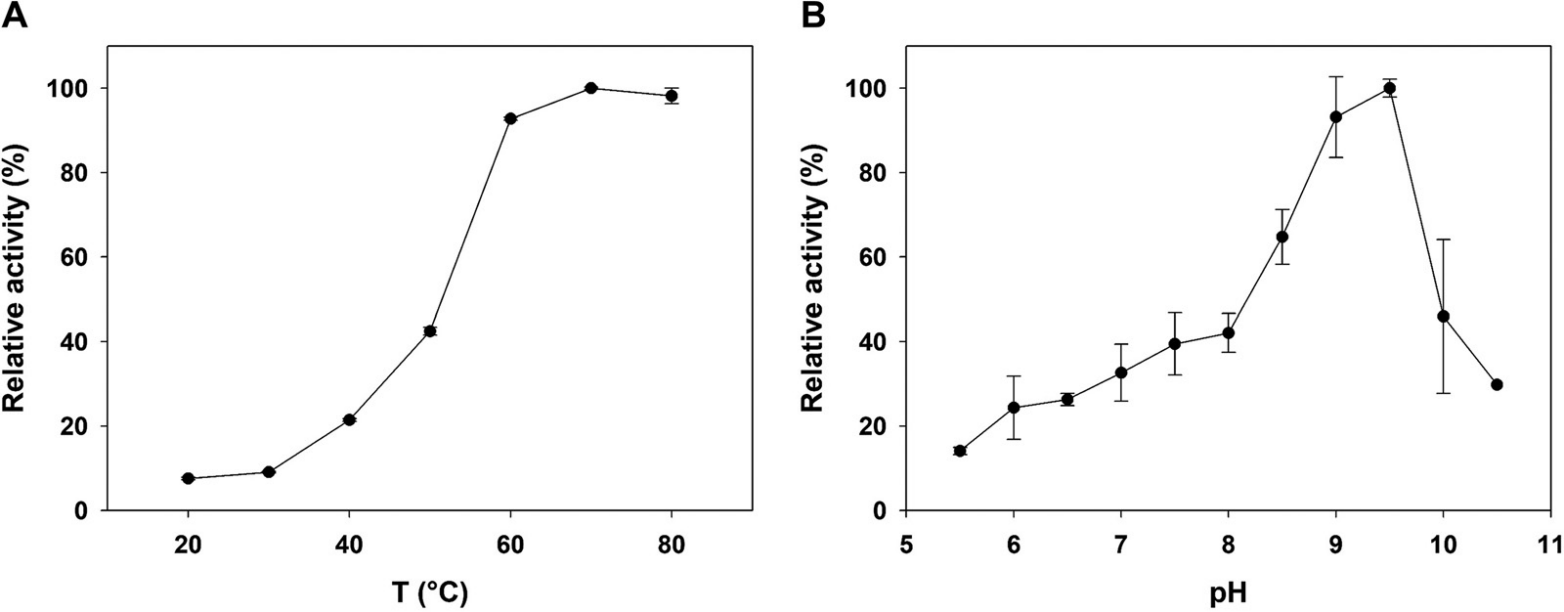
Dependence of *Ta*CPa2E activity on temperature and pH. (A) Temperature profile recorded with CDP-Glc (1 mM) in 100 mM MOPS buffer (pH 7.5) using HPLC (λ = 271 nm). The relative reference at 60°C corresponds to 13 mU/mg. (B) pH profile recorded with CDP-Glc (4 mM) at 60°C by high-performance anion exchange chromatography with pulsed amperometric detection (HPAEC-PAD). The enzyme activity at pH 9.5 (45.6 mU/mg) was used as a relative reference. All experiments were performed in triplicates.

We determined that when assayed at pH 7.5, *Ta*CPa2E exhibited an optimum temperature of ∼70°C (Fig. 5A). For further experiments, a lower temperature of 60°C was used to avoid sugar nucleotide degradation or enzyme inactivation. *Ta*CPa2E activity increased with pH up to a maximum value at pH 9.5 and declined rapidly at pH values above the optimum (Fig. 5B). A specific activity of 13 mU/mg protein was determined for the conversion of CDP-Glc into CDP-Man (pH 7.5 at 60°C).

### Sugar nucleotide synthesis.

CDP-Glc was initially prepared (∼50 mg; 45% yield) via the sucrose synthase reaction (43), converting sucrose and CDP directly into CDP-Glc and fructose. To facilitate the preparation of other substrates, we utilized a general, two-step strategy of anomeric phosphorylation and nucleotide coupling. The overall synthesis involved individual reactions done enzymatically, chemically if needed, or both in a suitable combination. While the synthesis of CDP-Glc (40 mg; ≥99% purity) was straightforward (∼75% conversion) in harnessing the promiscuity of known kinase (44) and pyrophosphorylase (45) enzymes (Scheme S1 and Fig. S8 to S11), the conversion of α-d-mannose 1-phosphate to CDP-Man required chemical nucleotide coupling to proceed in an ∼30% yield (Scheme S2 and Fig. S12 to S17). CDP-Man (∼1 mg) was nonetheless obtained at an excellent purity of 99% (Fig. S16 and S17).

In attempts to prepare CDP-Par, we used an “artificial biosynthesis” approach by following the route shown in Fig. 1. Starting from CDP-Glc, we performed a one-pot three-step transformation using isolated enzymes recombinantly produced in E. coli (Fig. S18). The Hallis group previously used an analogous procedure to synthesize CDP-Par as well as its 4-deoxy-4-fluoro analogue (19). Early studies had used cell extracts from Y. pseudotuberculosis to synthesize CDP-Par from CDP-Glc (41). Here, we encountered unexpected difficulties in getting the enzyme cascade reaction to run through. In search of the bottleneck, we analyzed each reaction separately. The 4,6-dehydration of CDP-Glc (5 to 20 mM) proceeded readily, and CDP-6-deoxy-d-*xylo*-hexopyranos-4-ulose (compound 2) (Fig. 1) was obtained in a quantitative yield (Fig. S19 and S20). Since lyophilization led to compound degradation, CDP-6-deoxy-d-*xylo*-hexopyranos-4-ulose was used in solution, as obtained from the reaction mixture after the removal of the enzyme. Incubation of the 4-keto-6-deoxy intermediate (1 to 20 mM) in the presence of the E1/E3 system did not give a product despite the full consumption of the NADH present (0.5 to 60 mM). When the synthase was additionally present, the intermediate was reduced to CDP-6-deoxy-d-glucose (CDP-6-deoxy-Glc). A preparative reaction of the 4-keto-6-deoxy intermediate (20 mM) using only the synthase in combination with NADH recycling gave the 6-deoxy product (∼3 mg; ∼96% purity) in an ∼98% yield (Scheme S3 and Fig. S20 and S22).

We extensively analyzed the E1/E3 system for 3-deoxygenation of CDP-6-deoxy-d-*xylo*-hexopyranos-4-ulose. E1 and E3 are both complex iron-sulfur proteins (46–49). E1 additionally requires a pyridoxamine 5′-phosphate cofactor (Fig. S23A) (50). We examined the recombinant enzymes from Y. pseudotuberculosis as well as the ones from *S.* Typhi. Enzymes with or without a His tag were used. Purified enzymes or enzymes from the E. coli cell extracts were used. His-tagged enzymes were purified by standard Ni^2+^ chelate chromatography or a combination of anion-exchange chromatography and gel filtration. To avoid elution with imidazole that could inactivate an otherwise active E1 enzyme, we also examined the E1 immobilized on an Ni^2+^ chelate matrix. The enzyme preparations showed no substrate conversion and/or product formation, although in all instances, the NADH was oxidized (Fig. S23B). E3 alone likewise oxidizes NADH (Fig. S23C and E). To exclude an E3 reaction uncoupled from the reaction of E1, the substrate mixture was made anoxic, which effectively suppressed NADH consumption (Fig. S23D) but failed to elicit combined E1/E3 activity. The absorbance spectrum of E1 (Fig. S23F) was consistent with the bound pyridoxamine cofactor that is required for activity. At this stage, therefore, we concluded that it was probably a dysfunctional iron-sulfur cluster in the enzymes as isolated (E1, E3, or both) that caused the interruption of intermolecular E3-to-E1 electron transport as the basis for combined E1/E3 function (51). Considering studies of other iron-sulfur proteins, we varied the conditions of recombinant production in E. coli (e.g., isopropyl-β-d-thiogalactopyranoside [IPTG] concentration of 50 to 1,000 μM, expression time of 1 to 20 h, expression temperature of 18°C to 37°C, and addition of trace metals). Solutions of isolated E1 and E3 were brownish (Fig. S24), as expected for iron-sulfur proteins. A detailed characterization of these iron-sulfur clusters to identify the reason for the lack of function was, however, beyond the scope of the current study.

As an alternative approach, we therefore considered the synthesis of CDP-d-tyvelose (CDP-Tyv) from commercial d-tyvelose. The sugar kinases NahK (44), GalKSpe4 (52), and hexokinase (combined with phosphoglucomutase) (19) did not promote anomeric phosphorylation. Chemical phosphorylation according to previously reported protocols (53, 54) proceeded in an ∼90% yield; however, subsequent nucleotide coupling (chemical or enzymatic) did not proceed. We considered the aggregate evidence of failed attempts at CDP-Par or CDP-Tyv of potential importance to others in the field for it to be reported here in brief.

To nonetheless examine a substrate featuring deoxygenation at C-3, we prepared CDP-3-deoxy-d-glucose (CDP-3-deoxy-Glc) using the enzymatic route of 3-deoxy-Glc phosphorylation from ATP and nucleotide coupling from CMP (Scheme S4). The desired product was obtained at 84% purity and in a good (60%) yield from commercial starting material (Fig. S25 to S27).

### Kinetic characterization of *Ta*CPa2E.

*Ta*CPa2E-catalyzed C-2 epimerization is a freely reversible reaction in which CDP-Glc and CDP-Man are converted into one another, proceeding through a transient C-2 keto intermediate (19). Initial rates were recorded in the forward and reverse directions of the reaction using CDP-Glc and CDP-Man as the substrates, respectively. Substrate consumption was linear with time in the early reaction phase. There was a close balance between the substrate utilized and the product formed, effectively ruling out the release of the 2-keto intermediate from enzyme-NADH to a significant degree.

**FIG 6:**
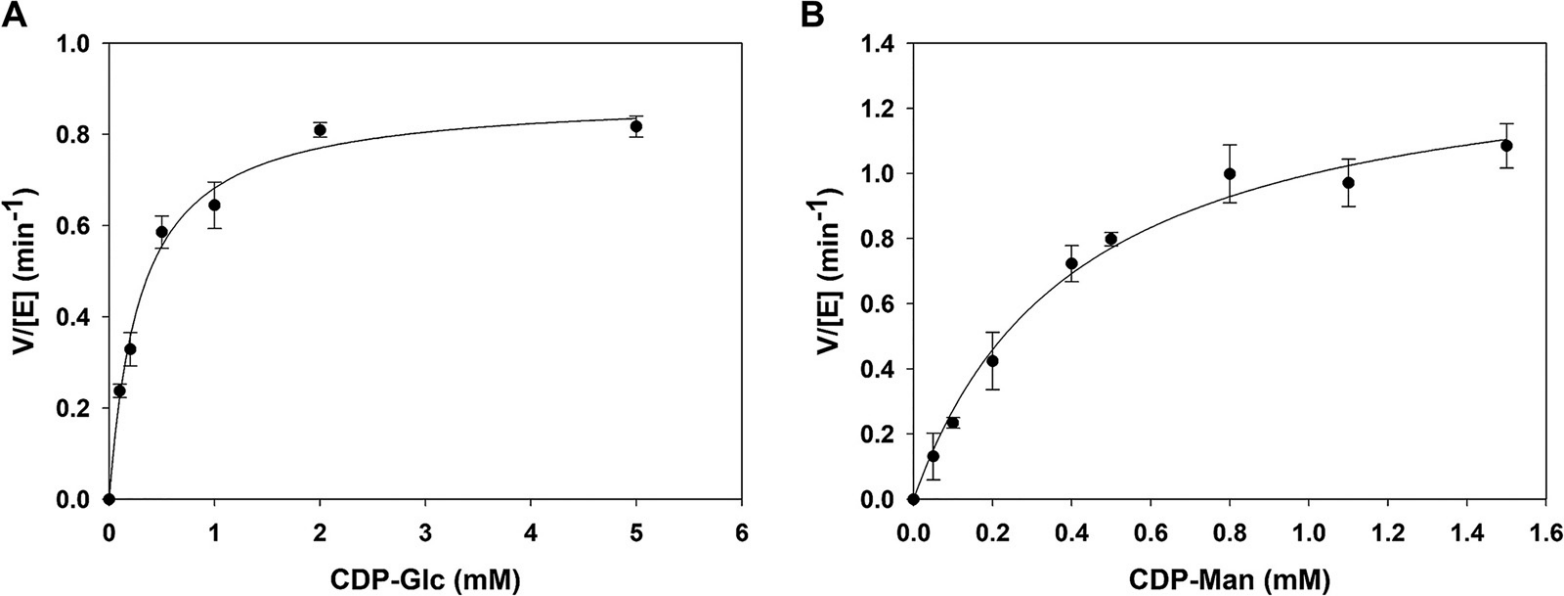
Kinetic characterization of *Ta*CPa2E for interconversion of CDP-Glc (A) and CDP-Man (B). Initial reaction rates (V) divided by the molar enzyme concentration [E] are shown. Reactions were performed in triplicates at 60°C and pH 7.5 and analyzed by HPLC (λ = 271 nm).

Kinetic parameters (*k*_cat_ and *K_m_*) were obtained from nonlinear fits of the data as shown in Fig. 6A and B. The *K_m_* values were 0.32 ± 0.02 mM for CDP-Glc and 0.31 ± 0.01 mM for CDP-Man, and the *k*_cat_ values were 0.90 ± 0.01 min^−1^ for CDP-Glc and 1.40 ± 0.03 min^−1^ for CDP-Man. The *K*_eq_ from the Haldane relationship (55) was calculated using the catalytic efficiencies (*k*_cat_/*K_m_*) for forward (2.8 mM^−1 ^min^−1^) and reverse (4.5 mM^−1 ^min^−1^) epimerization, resulting in a *K*_eq_ of 0.64. The kinetically determined *K*_eq_ is in excellent accordance with the directly measured equilibrium constant (0.67). The internal consistency of the kinetic parameters for forward and reverse directions of the reaction is thus shown. The equilibrium of CDP-Glc C-2 epimerization differs from that of C-4 epimerization of UDP-Glc (*K*_eq_ = 3.5) in E. coli (56). The thermodynamics of sugar nucleotide epimerization may warrant further study for a better understanding of the equilibrium positions associated with these reactions.

### Substrate specificity of *Ta*CPa2E.

The turnover rates for the *Ta*CPa2E-catalyzed epimerization of nucleotide variants of activated d-glucose were determined. Pyrimidine nucleotides (CDP, UDP, and TDP) were preferred ≥6.5-fold over purine nucleotides (GDP and ADP). The order of reactivity was CDP-Glc (15 × 10^−3^ s^−1^; 22.8 mU/mg), UDP-Glc (2.5 × 10^−3^ s^−1^; 3.8 mU/mg), TDP-Glc (1.10 × 10^−3^ s^−1^; 1.67 mU/mg), GDP-Glc (0.16 × 10^−3^ s^−1^; 0.25 mU/mg), and ADP-Glc (0.09 × 10^−3^ s^−1^; 0.13 mU/mg). We confirmed that epimerization occurred at C-2, generating a d-mannosyl product, in each case. The nucleotide promiscuity of *Ta*CPa2E is remarkable considering the high degree of substrate specificity that SDR-type epimerases often exhibit (57, 58).

We then examined CDP-6-deoxy-Glc, CDP-3-deoxy-Glc, and CDP-6-deoxy-d-*xylo*-hexopyranos-4-ulose as the substrates of *Ta*CPa2E (Schemes S5 to S7) The enzyme was inactive (≤0.1% of the activity with CDP-Glc) with CDP-6-deoxy-d-*xylo*-hexopyranos-4-ulose and, interestingly, also with CDP-3-deoxy-Glc. The specific activity with CDP-6-deoxy-Glc (62 mU/mg) was about 5 times that with CDP-Glc (13 mU/mg) at a 1 mM substrate concentration.

**FIG 7:**
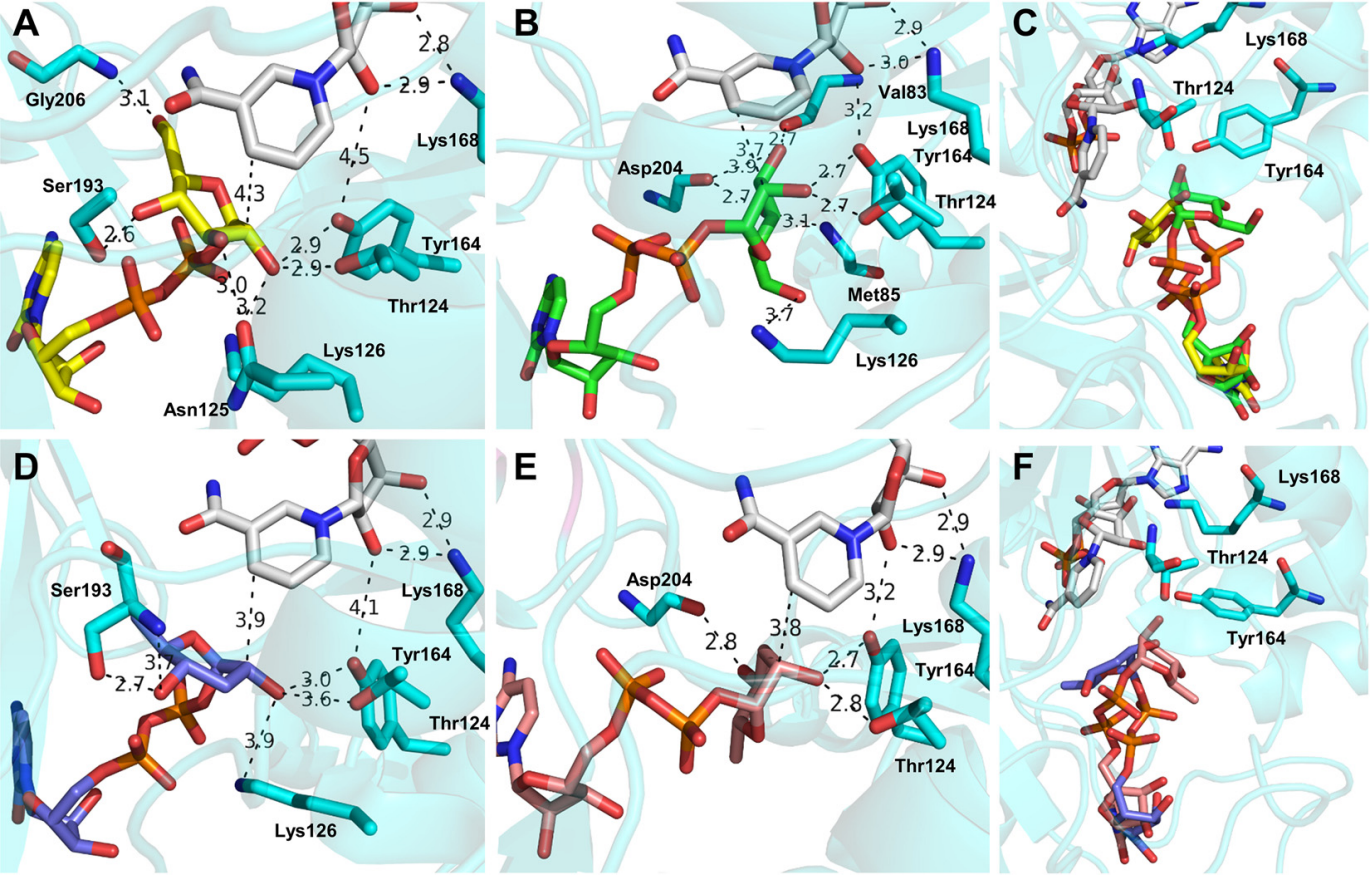
Binding of CDP-Glc, CDP-Man, CDP-Par, and CDP-Tyv to *Ta*CPa2E analyzed by structure modeling and ligand docking. The docking poses of CDP-Glc (A), CDP-Man (B), CDP-Glc/CDP-Man (C), CDP-Par (D) CDP-Tyv (E), and CDP-Par/CDP-Tyv (F) with tightly bound NAD^+^ and the main active-site residues are shown. Atom coloring is used: C atoms are shown in yellow (CDP-Glc), green (CDP-Man), light blue (CDP-Par), salmon (CDP-Tyv), cyan (enzyme), and gray (NAD^+^). Relevant distances are indicated in angstroms. For further discussion, see the text.

To obtain a molecular interpretation of the particular substrate specificity of *Ta*CPa2E, we docked CDP-Glc and CDP-Man, as well as CDP-Par and CDP-Tyv (Fig. 7), into a structure model of the enzyme bound with NAD^+^. The homology model was generated from the 1.5-Å-resolution crystal structure of stTyvE-NAD^+^ in complex with CDP (36). The two enzymes are 65% identical in sequence, and the Yasara-built model of *Ta*CPa2E was well defined. The obtained docking poses were regarded as plausible with respect to enzyme catalysis for each involved positioning of the main active-site residues (Thr124, Tyr164, and Lys168) to support oxidation at the sugar C-2 according to the canonical SDR mechanism. The C-2 OH was coordinated tightly by Tyr164 (the catalytic base), and Thr124 contributed to its orientation (Fig. 7A, B, D, and E). The C-2 was positioned reasonably (3.7- to 4.3-Å distance) for hydride transfer to the nicotinamide C-4 of NAD^+^. Lys168 was placed properly to establish a proton relay from Tyr164 via the ribose hydroxyls of NAD^+^. Accommodation of the sugar nucleotide C-2 epimers (Fig. 7A, B, D, and E) in the enzyme binding pocket involved a markedly different orientation of the sugar residue. Superimposition of the docking poses (Fig. 7C and F) shows that with the nucleotide part fixed in place, complex bond rotations within the pyrophosphate moiety were required to promote the change in orientation. Interestingly, Glc and Par, as well as their corresponding C-2 epimers, were accommodated, with their pyranose rings adopting an undistorted ^4^C_1_ conformation. Despite the preliminary stage of this analysis, the docking results support enzymatic C-2 epimerization via rotation/flipping of a transient 2-keto-hexose intermediate to enable nonstereospecific reduction by enzyme-NADH.

The docking results further suggest enzyme residues with an auxiliary role in catalysis and contributing to substrate specificity. Ser193 (side chain) and Asp204 (main-chain carbonyl) are key residues to form hydrogen bonds with the 4-OH of substrates in the d-*gluco* (Glc and Par) and d-*manno* (Man and Tyv) configurations, respectively (Fig. 7A, B, D, and E). The lack of activity with CDP-6-deoxy-d-*xylo*-hexopyranos-4-ulose is explainable by the partial (Ser193) and complete (Asp204) loss of bonding at substrate position C-4 along the catalytic reaction path. In Par and Tyv, the deoxygenated positions C-3 and C-6 appear to have no direct role in substrate binding, as shown in Fig. 7D to F. Using C-3 and C-6 deoxygenation one at a time, we assessed the roles of the two hydroxy groups individually. The CDP-6-deoxy-Glc substrate shows that the 6-OH is likely not important for activity, consistent with the docking results that indicate only weak interactions with the enzyme (bonding distance of ≥3.0 Å) (Fig. 7A and B). Asn125 (side chain; 3.0 Å) and Val83 (main-chain carbonyl; 2.7 Å) form a hydrogen bond with the 3-OH in CDP-Glc and CDP-Man, respectively (Fig. 7A and B). Evidence showing that CDP-3-deoxy-Glc is completely inactive indicates that the individual substitution of the 3-OH has a more globally disruptive effect on the substrate positioning than one would expect from the local disruption of a hydrogen bond at C-3. Therefore, the assumed activity of *Ta*CPa2E with the 3,6-dideoxygenated CDP-Par could not be explained by independent (i.e., additive) contributions of the individual deoxygenations but would necessitate that the two are cooperative in a way not intuitive from the docking analysis. Our results show that substrate recognition by the epimerase is well aligned to the order of the steps in the biosynthetic pathway (Fig. 1), ruling out that C-2 epimerization can happen at the level of intermediates prior to CDP-Par. The *k*_cat_ of *Ta*CPa2E for CDP-Glc (∼1 min^−1^) is lower, but not dramatically so, than the *k*_cat_ of ypTyvE with CDP-Par (22 ± 1 min^−1^) (19). The sugar nucleotide poses obtained for CDP-Glc and CDP-Man fulfill the requirements for oxidation/reduction-based C-2 epimerization by *Ta*CPa2E.

**FIG 8:**
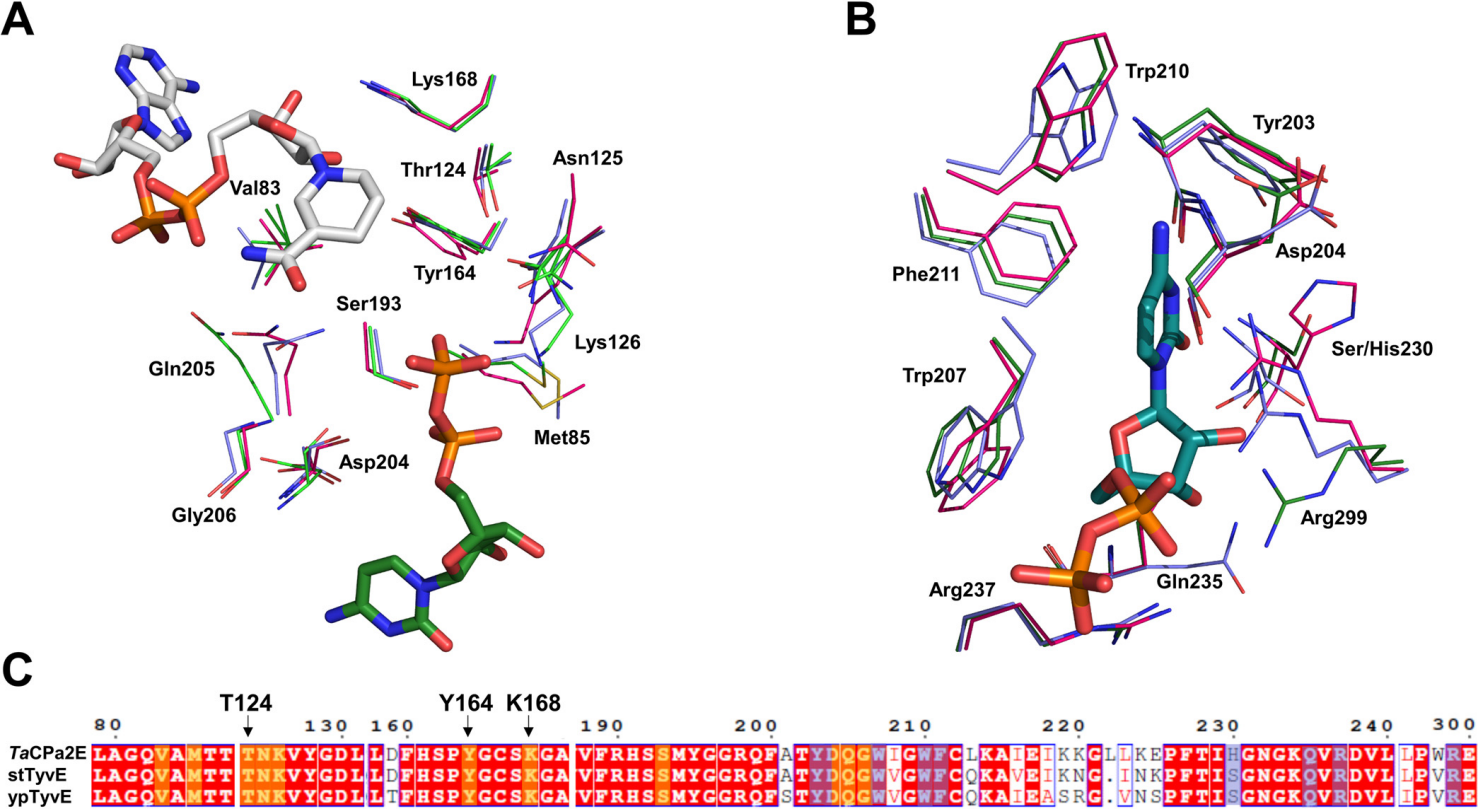
Partial structural and sequence alignment of *Ta*CPa2E, ypTyvE, and stTyvE. (A and B) Sugar binding pocket (A) and nucleotide binding site (B). Atom coloring is used: C atoms are depicted in pink (*Ta*CPa2E), green (stTyvE), light blue (ypTyvE), turquoise (CDP), and gray (NAD^+^). (C) Partial sequence alignment of *Ta*CPa2E, ypTyvE, and stTyvE. Residues framing the sugar binding pocket and nucleotide binding site are highlighted in yellow and light blue, respectively.

In addition, a homology model of ypTyvE was generated based on the crystal structure of stTyvE. In order to determine the residues framing the enzymes’ active sites, *Ta*CPa2E, stTyvE, and ypTyvE were aligned based on sequence and structure (Fig. 8). The residues in the sugar binding pocket, as suggested by the docking analysis, are fully conserved (Fig. 8A and C). Analyzing the binding pocket for CDP (Fig. 8B and C), we find that the interacting enzyme residues are highly conserved as well (≥88%), deviating merely at position 230, comprising Ser in ypTyvE/stTyvE and His in *Ta*CPa2E. Similar to Tyr203, amino acids in these two positions are interacting with the cytosine ring via their main-chain carbonyl oxygen, or the peptide amino group, and hence can be considered to be interchangeable. Combining the findings for the X-ray structure of stTyvE for nucleotide recognition with the homology models generated, important residues for positioning cytidine, such as Trp207, Trp210, and Phe211, are conserved in stTyvE, ypTyvE, and *Ta*CPa2E. The same applies to Arg237 and Arg299, involved in phosphate positioning, and Asp204 and Gln235, interacting with ribose. Due to a surprisingly high sequence conservation, the promiscuity of *Ta*CPa2E cannot be solely explained by either amino acid composition or diverging structural features.

In summary, we have discovered a new TyvE-like enzyme from Thermodesulfatator atlanticus that catalyzes C-2 epimerization of CDP-Glc. The enzyme represents a new subgroup of SDR-type C-2 epimerases that show unique substrate specificity. *Ta*CPa2E was characterized biochemically, and the unprecedented enzymatic interconversion of CDP-Glc and CDP-Man was analyzed in detail. Elements of substrate binding recognition crucial for activity with CDP-Glc were suggested from a combination of experimental (activity studies of synthetic analogues of CDP-Glc) and computational (molecular docking) analyses. The epimerization reaction appears to involve complex bond rotations within the substrate’s pyrophosphate group to enable flipped positions of the transient 2-keto intermediate for nonstereospecific reduction. Catalytic oxidation-reduction coordinated with molecular rotation in the enzyme’s active site is a general mechanistic principle of sugar nucleotide-modifying epimerases of the SDR superfamily (10, 19, 21, 58, 59).

## MATERIALS AND METHODS

### Materials.

Nucleotides and sugar nucleotides were obtained from Carbosynth (Compton, Berkshire, UK). d-Tyvelose was obtained from Santa Cruz Biotechnology, Inc. (Dallas, TX, USA). Deuterium oxide (99.96% ^2^*H*) was obtained from Euriso-Top (Saint-Aubin, France). Toyopearl SuperQ-650M was obtained from Tosoh Bioscience (Tokyo, Japan), and Sephadex G-10 resin was obtained from GE Healthcare (Vienna, Austria). Fe(III)-coated resins were obtained from EnginZyme (Stockholm, Sweden). All other chemicals and reagents were of the highest available purity. E. coli BL21(DE3) competent cells were prepared in-house. A GeneJET plasmid miniprep kit (Thermo Scientific, Waltham, MA, USA) was used for plasmid DNA isolation.

### Enzymes.

Genes codon optimized for expression in E. coli BL21(DE3) were obtained by GeneArt gene synthesis (Thermo Fisher). A pET21a expression vector was used unless stated otherwise. For details of the expressions done, see the supplemental material. The protein concentration was determined by using a Nanodrop instrument.

### *Ta*CPa2E.

A C-terminally His-tagged version of *Ta*CPa2E (NCBI accession number WP_022854415.1) was used. Cells grown at 37°C in terrific broth (TB) medium (ampicillin at 100 μg/ml) were induced with 100 μM IPTG at 18°C for 20 h. *Ta*CPa2E was isolated from cell lysates by immobilized Ni^2+^ chromatography (see the methods in the supplemental material). The purity and molecular size of *Ta*CPa2E were shown by SDS-PAGE.

### Enzymes for general sugar nucleotide synthesis.

The enzymes used were *N*-acetylhexosamine 1-kinase (NahK) from Bifidobacterium longum (EC 2.7.1.162), galactokinase (GalKSpe4) from Streptococcus pneumoniae (EC 2.7.1.6), inorganic pyrophosphatase (iPPase) from E. coli (EC 3.6.1.1), UDP-glucose pyrophosphorylase (UGPase) from Bifidobacterium longum (EC 2.7.7.9), and sucrose synthase (AcSuSy) from Acidithiobacillus caldus (EC 2.4.1.13). The supplemental material describes production in E. coli and purification of the enzymes. Pyruvate kinase from rabbit muscle (EC 2.7.1.40), glucose oxidase from Aspergillus niger (EC 1.1.3.4), and catalase from bovine liver (EC 1.11.1.6) were obtained from Sigma-Aldrich. Formate dehydrogenase from Candida boidinii (EC 1.17.1.9) was prepared in E. coli as reported previously (60). It was used as a freeze-dried whole-cell preparation.

### Enzymes for CDP-Par synthesis.

The genes for CDP-d-glucose 4,6-dehydratase (DH), E1, E3, and CDP-d-paratose synthase (PS) (Fig. 1) were from *Salmonella* Typhi. Genes for E1 and E3 were also from Yersinia pseudotuberculosis. All genes for *S*. Typhi enzymes had a C-terminal His tag. The Y. pseudotuberculosis enzymes were His tagged (N or C terminus) or untagged. Expression was done in LB or TB medium (see the supplemental material), which in the case of E1 or E3 production was optionally supplemented with (NH_4_)_4_Fe(SO_4_)_2_ at up to 200 μM. Tagged enzymes were isolated by standard metal affinity chromatography. Untagged enzymes were prepared by anion-exchange chromatography and gel filtration. Carefully degassed buffers were used when purifying E1 and E3.

### Enzyme-bound nicotinamide cofactor.

Methanol (MeOH) (30 μl) was added to the enzyme (33.7 mg/ml *Ta*CPa2E; 858.3 μM; 30 μl) and incubated at 30°C for 3 h without agitation. Controls containing NAD^+^ or NADH without the enzyme were incubated identically in the absence and presence of MeOH. Samples were centrifuged for 1 h at 21,130 × *g*. The enzyme pellet was resuspended in 30 μl of 6 M urea and subjected to HPLC analysis. Experiments were done in duplicates.

### Sugar nucleotide synthesis.

CDP-Glc (see Scheme S1 in the supplemental material), CDP-Man (Scheme S2), CDP-6-deoxy-d-*xylo*-hexopyranos-4-ulose (compound 2) (Fig. 1), CDP-6-deoxy-Glc (Scheme S3), and CDP-3-deoxy-Glc (Scheme S4) were prepared by suitable combinations of chemical and enzymatic reactions. The supplemental material (Fig. S8 to S27) provides details of the synthesis conditions, isolation, and NMR structural characterization of the products. GDP-d-Glucose was prepared from sucrose and nucleoside diphosphate using sucrose synthase (see the supplemental material).

Attempts to synthesize CDP-Par are summarized in the supplemental material. CDP-6-Deoxy-d-*xylo*-hexopyranos-4-ulose obtained from CDP-Glc (1 to 20 mM) (Fig. 1) was incubated with E1 and E3 to promote deoxygenation at C-3. Various conditions were examined, including reactions with His-tagged or untagged enzymes, anoxic reactions, reactions in the presence of an immobilized enzyme, or reactions with the regeneration of NADH (see the supplemental material). As an alternative, synthesis started from d-tyvelose via chemical anomeric phosphorylation and coupling (Fig. S28).

### Sugar nucleotide isolation.

Anion-exchange chromatography (Toyopearl SuperQ‐650M; Tosoh Bioscience, Tokyo, Japan) was used for separation. Gel filtration (Sephadex G‐10; GE Healthcare) was used for desalting. Protocols are provided in the supplemental material. Concentration was performed with a rotary evaporator (Laborota 4000; Heidolph, Schwabach, Germany) at 40°C at 2 × 10^3^ Pa. Freeze-drying was done with a Christ Alpha 1-4 lyophilizer (B. Braun Biotech International, Melsungen, Germany). To prevent degradation of the sugar nucleotides, the sample pH was adjusted to 4.

### Kinetic characterization and substrate specificity. (i) Kinetic parameters.

Substrate solutions (CDP-Glc, 0.1 to 20 mM; CDP-Man, 0.05 to 1.5 mM) were prepared based on a molar extinction coefficient of 9.3 mM^−1^ cm^−1^ at 271 nm. A 100 mM morpholinepropanesulfonic acid (MOPS) buffer (pH 7.5) was used. Reactions were carried out at 60°C without agitation (thermomixer comfort; Eppendorf AG, Hamburg, Germany). Purified *Ta*CPa2E was used at 0.05 to 0.2 mg/ml (1.3 to 5 μM) to give a substrate conversion of 0.3 to 18% suitable for initial rate analysis. The final volume was between 60 μl and 100 μl. Control reaction mixtures lacked the enzyme. Reactions were quenched after 20 min by adding a 30-μl sample to 70 μl acetonitrile (ACN)-H_2_O (2:1). Note that substrate consumption and product release were shown to be linear with reaction time. Samples were centrifuged for 30 min at 21,130 × *g*. Supernatants from centrifuged samples (30 min at 21,130 × *g*) were analyzed by HPLC. Experiments were performed in triplicates. Kinetic parameters (*V*_max_ and *K_m_*) were determined from nonlinear fits of initial rates dependent on the substrate concentration. The enzyme catalytic constant (*k*_cat_) was obtained from the relationship *V*_max_ = *k*_cat_[E], where [E] is the molar enzyme concentration determined from the protein mass concentration and the molecular mass of the enzyme subunit (39.3 kDa).

### (ii) Substrate specificity, pH, and temperature dependence of activity.

Specific activities for nucleotide (CDP, GDP, ADP, and UDP)-activated glucoses were determined at 60°C. A 100 mM MOPS buffer (pH 7.5) was used. The substrate concentration was 5 mM. Purified *Ta*CPa2E was used at 1 mg/ml (25.4 μM). After a suitable reaction time to give 20 to 25% conversion, the enzyme was acid/heat inactivated, and the sugar nucleotides were hydrolyzed. Hydrolysis involved a 20-fold dilution of the sample in 100 mM sodium acetate (NaOAc) and incubation at 95°C for 1 h. The released Glc and Man were analyzed by HPAEC-PAD. One enzyme unit equals 1 μmol Man/min under the conditions used.

The pH-activity profile was determined at 60°C. Purified *Ta*CPa2E (0.5 mg/ml; 12.7 μM) was used at a saturating CDP-Glc concentration (4 mM). The buffers used were 100 mM MOPS (pH range, 5.5 to 7.5), Gly-Gly (pH range, 7.5 to 8.5), morpholineethanesulfonic acid (MES) (pH range, 8.5 to 9.5), and 3-(cyclohexylamino)-2-hydroxy-1-propanesulfonic acid (CAPSO) (pH range, 9.5 to 10.5). Samples (≤20% conversion) were hydrolyzed and analyzed by HPAEC-PAD. The temperature-activity profile was recorded with 1 mM CDP-Glc using 0.5 mg/ml *Ta*CPa2E in 100 mM MOPS buffer (pH 7.5). All experiments were performed in triplicates.

### (iii) *In situ* proton NMR analysis.

*In situ* proton NMR analysis was performed in D_2_O buffer (50 mM K_2_HPO_4_/KH_2_PO_4_, pD 7.5 [pD is the pH meter reading plus 0.4]). The reaction mixture contained 15.3 μM (0.6 mg/ml) *Ta*CPa2E and 4 mM CDP-Glc. Data acquisition was performed at 60°C on a Varian Inova 500-MHz NMR spectrometer (Agilent Technologies, Santa Clara, CA, USA) every 10 min from the enzyme addition. VNMRJ 2.2D software was used for the measurements. ^1^H NMR spectra (499.98 MHz) were recorded on a 5-mm indirect-detection pulsed-field-gradient (PFG) probe with presaturation of the water signal by a shaped pulse. The spectra were analyzed using MestReNova 16.0 (Mestrelab Research, SL).

### Analytics. (i) HPLC.

The reaction sample (30 μl) was mixed with 70 μl ACN-H_2_O (2:1) and centrifuged (30 min at 21,130 × *g*). Supernatants were analyzed on a Shimadzu HPLC system equipped with a Kinetex C_18_ analytical HPLC column (150- by 4.6-mm, 5-μm Evo C_18_ 100-Å column; Phenomenex, Aschaffenburg, Germany) with UV detection at 271 nm. Injection volumes were between 5 and 20 μl. Unless stated otherwise, isocratic flow (1 ml/min) was used at 40°C, with the mobile phase composed of potassium phosphate buffer (pH 5.9) containing tetrabutylammonium bromide (98%) (solvent A) and methanol (2%) (solvent B).

### (ii) Capillary zone electrophoresis.

Samples prepared as described above for HPLC were used. Capillary zone electrophoresis (CE) was performed at 50°C on an HP 3D CE system (Hewlett Packard, Palo Alto, CA, USA) equipped with an extended light path fused-silica capillary (5.6 mm by 56 cm) from Agilent Technologies and a diode array detector (λ = 271 nm and 340 nm). Sodium tetraborate buffer (20 mM; pH 9.3) was used. The capillary was preconditioned with H_2_O (5 min), 0.1 M NaOH (5 min), H_2_O (5 min), and 20 mM sodium tetraborate (8 min). The sample was injected by pressure (5 × 10^3^ Pa for 5 s or 10 s) and analyzed with a voltage of up to 30 kV for 12 min.

### (iii) HPAEC-PAD.

A Dionex ICS-3000 system (CarboPac PA20 column, 3 by 150 mm; Thermo Fischer Scientific) equipped with a pulsed amperometric detector was used. An isocratic flow (0.5 ml/min for 15 min) of 100 mM NaOH (12%) and doubly distilled water (88%) was used.

### (iv) Thin-layer chromatography.

The eluent was 1-butanol–acetic acid–doubly distilled H_2_O in a volume ratio of 2/1/1. Standard silica plates (TLC silica gel 60 F_254_; Merck, Darmstadt, Germany) were used. The staining solution was thymol–ethanol (EtOH)–H_2_SO_4_ in a 0.5:95:5 (wt/vol/vol) ratio.

### (v) Bioinformatic analysis, structure modeling, and docking.

The Domain Enhanced Lookup Time Accelerated BLAST tool (NCBI, Bethesda MD, USA) was used for motif-based sequence retrieval. Multiple-sequence alignments and phylogenetic analyses were performed using MEGA X (61, 62) and ESPript (63). The evolutionary history was inferred by using the maximum likelihood method and a Jones-Taylor-Thornton (JTT) matrix-based model. The tree with the highest log likelihood (−9,467.60) is shown. The initial tree(s) for the heuristic search was obtained automatically by applying the Neighbor-Join and BioNJ algorithms to a matrix of pairwise distances estimated using the JTT model and then selecting the topology with the superior log-likelihood value. The tree is drawn to scale, with branch lengths measured in the number of substitutions per site. This analysis involved 22 amino acid sequences. There were a total of 398 positions in the final data set.

The PyMOL molecular graphics system (open source; Schrödinger, LLC) was used for structural alignments. Docking experiments and modeling were performed using Yasara (Yasara Biosciences GmbH, Vienna, Austria). The homology models of *Ta*CPa2E and ypTyvE were built upon the crystal structure of stTyvE (PDB accession number 1ORR) using the homology modeling macro. Local ligand docking was carried out with Autodock Vina (64) using standard parameters. The binding energies of CDP-Glc and CDP-Man are 9.41 and 8.93 kcal mol^−1^ with dissociation constants of 126.98 and 287.01 nM, respectively. The Z-scores for the homology models of *Ta*CPa2E and ypTyvE are −0.023 and −0.215, respectively.

## Supplementary Material

Supplemental file 1
