# Supplementary material for: Expanding the Enzyme Repertoire for Sugar Nucleotide Epimerization: the CDP-Tyvelose 2-Epimerase from Thermodesulfatator atlanticus for Glucose/Mannose Interconversion
Source: Appl Environ Microbiol. 2021 Jan 29;87(4):e02131-20. doi: 10.1128/AEM.02131-20 (PMC7851689; doi:10.1128/AEM.02131-20)
Supplement: Supplemental file 1 [file AEM.02131-20-s0001.pdf]

SUPPLEMENTARY INFORMATION

Expanding the enzyme repertoire for sugar nucleotide epimerization: the CDP-tyvelose 2-epimerase from *Thermodesulfatator atlanticus* for glucose/mannose interconversion

Christian Rapp[a], Stevie van Overtveldt[b], Koen Beerens[b], Hansjörg Weber[c], Tom Desmet[b,d], and Bernd Nidetzky[a,d,#]

[a] Institute of Biotechnology and Biochemical Engineering, Graz University of Technology, NAWI Graz, 8010 Graz, Austria

[b] Centre for Synthetic Biology, Department of Biotechnology, Ghent University, 9000 Ghent, Belgium

[c] Institute of Organic Chemistry, Graz University of Technology, NAWI Graz, Stremayrgasse 9, 8010 Graz, Austria

[d] Austrian Centre of Industrial Biotechnology (acib), 8010 Graz, Austria

# Corresponding author: bernd.nidetzky@tugraz.at

Christian Rapp and Stevie van Overtveldt contributed equally to this work.

Table of contents

Enzyme characterization **3**

Sugar nucleotide synthesis I **11**

CDP-α-D-glucose **11**

CDP-α-D-mannose **14**

Sugar nucleotide synthesis II *CDP-α-D-glucose derivatives* **19**

Enzymes for CDP-α-D-paratose synthesis **19**

CDP-6-deoxy-D-*xylo*-hexopyranos-4-ulose **20**

CDP-6-deoxy-α-D-glucose **21**

CDP-3,6-dideoxy-D-*xylo*-hexopyranos-4-ulose (attempted) **23**

CDP-3-deoxy-α-D-glucose **24**

Experimental procedures (addition to the main text) **28**

Gene expression and protein purification **28**

CDP-α-D-paratose synthesis (attempted) **30**

CDP-α-D-tyvelose synthesis (attempted) **32**

GDP-α-D-glucose synthesis **32**

Sugar nucleotide isolation **33**

References **34**

Enzyme characterization

DNA sequence of *Ta*CPa2E

ATGAAATACCTGATTACCGGTGGTTGTGGTTTTCTGGGTAGCAATCTGGCAAGCGAAGTTCTGCGTAA
ACGTGAAGAACTGATCATCTTCGATAACCTGTATCGCATTGGCAGCTATGAAAATCTGGAATGGCTGA
AACAGCAGGGTGATTTTCGTTTTGTTCATGGCGATATTCGTAACCGCGAAGATATTGAGAAAGTGATC
AAAGAAGAAAAACCGGACGTTATCTTTCATCTGGCAGGTCAGGTTGCAATGACCACCAGTATTGAAAA
TCCGCGTCTGGATTTTGAAGTTAATGCACTGGGCACCTTTAATCTGCTGGATGCAGTTCGTAAATATAG
TCCGGAAAGCATCATTATCTATAGCAGCACCAATAAAGTGTATGGTGATCTGGGTTGGGTTCGTTATG
AAGAAACCGAAACCCGTTATATTGCACCGGATTTTCCGAATGGTTTTCCGGAATCAATTCCGCTGGATT
TTCATTCACCGTATGGTTGTAGCAAAGGTGCAGCAGATCAGTATATGCTGGATTTCTGTCGCATTTATG
GCATTAAAACCGTGGTTTTTCGTCACAGCAGCATGTATGGTGGTCGTCAGTTTGCAACCTATGATCAGG
GTTGGATTGGTTGGTTTTGTCTGAAAGCCATCGAAATCAAAAAAGGCCTGCTGAAAGAACCGTTTACC
ATTCATGGTAATGGCAAACAGGTTCGTGATGTTCTGTATGCCGATGATATGATCGAGCTGTATTTCAAA
ACCATCGAAAACATCGAGAAAGCCAAAGGTGAAGCCTTTAACATTGGTGGTGGTATGGAAAATAGCC
TGAGCCTGCTGGAACTGTTTTCACTGCTGGAAAAAGAACTGGATATCAAAATGCGCTACAAAAAACTG
CCGTGGCGTGAAAGCGATCAGAAAATCTTTGTTGCAGATATTCGCAAAGCCCAGAAACTGATTGGCTG
GCAGCCGAAAGTTAGCAAATACAATGGTATTCAGAAAATGCTGGAATGGATCAAAAAACTGGTGCTC
GAGCACCACCACCACCACCACTGA

Amino acid sequence of *Ta*CPa2E

M K Y L I T G G C G F L G S N L A S E V L R K R E E L I I F D N L Y R I G S Y E N L E
W L K Q Q G D F R F V H G D I R N R E D I E K V I K E E K P D V I F H L A G Q V A M T
T S I E N P R L D F E V N A L G T F N L L D A V R K Y S P E S I I I Y S S T N K V Y G D
L G W V R Y E E T E T R Y I A P D F P N G F P E S I P L D F H S P Y G C S K G A A D Q
Y M L D F C R I Y G I K T V V F R H S S M Y G G R Q F A T Y D Q G W I G W F C L K A
I E I K K G L L K E P F T I H G N G K Q V R D V L Y A D D M I E L Y F K T I E N I E K A
K G E A F N I G G G M E N S L S L L E L F S L L E K E L D I K M R Y K K L P W R E S D
Q K I F V A D I R K A Q K L I G W Q P K V S K Y N G I Q K M L E W I K K L V H H H H
H H **Stop**

HisTag

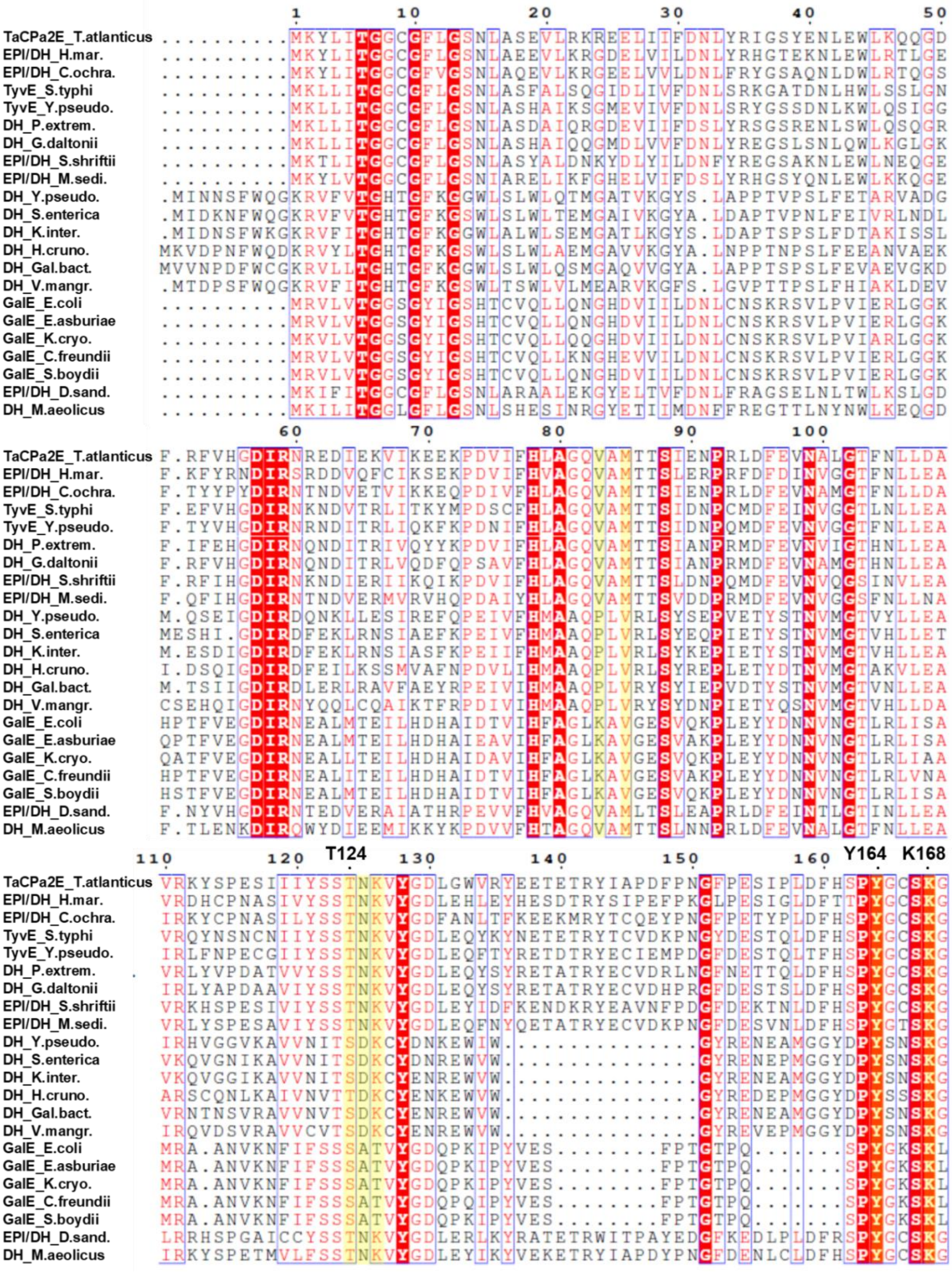
TaCPa2E_T.atlanticus
EPI/DH_H.mar.
EPI/DH_C.ochra.
TyvE_S.typhi
TyvE_Y.pseudo.
DH_P.extrem.
DH_G.daltonii
EPI/DH_S.shriftii
EPI/DH_M.sedi.
DH_Y.pseudo.
DH_S.enterica
DH_K.inter.
DH_H.cruno.
DH_Gal.bact.
DH_V.mangr.
GalE_E.coli
GalE_E.asburiae
GalE_K.cryo.
GalE_C.freundii
GalE_S.boydii
EPI/DH_D.sand.
DH_M.aeolicus
1
10
20
30
40
50
60
70
80
90
100
110
120
T124
130
140
150
160
Y164
K168

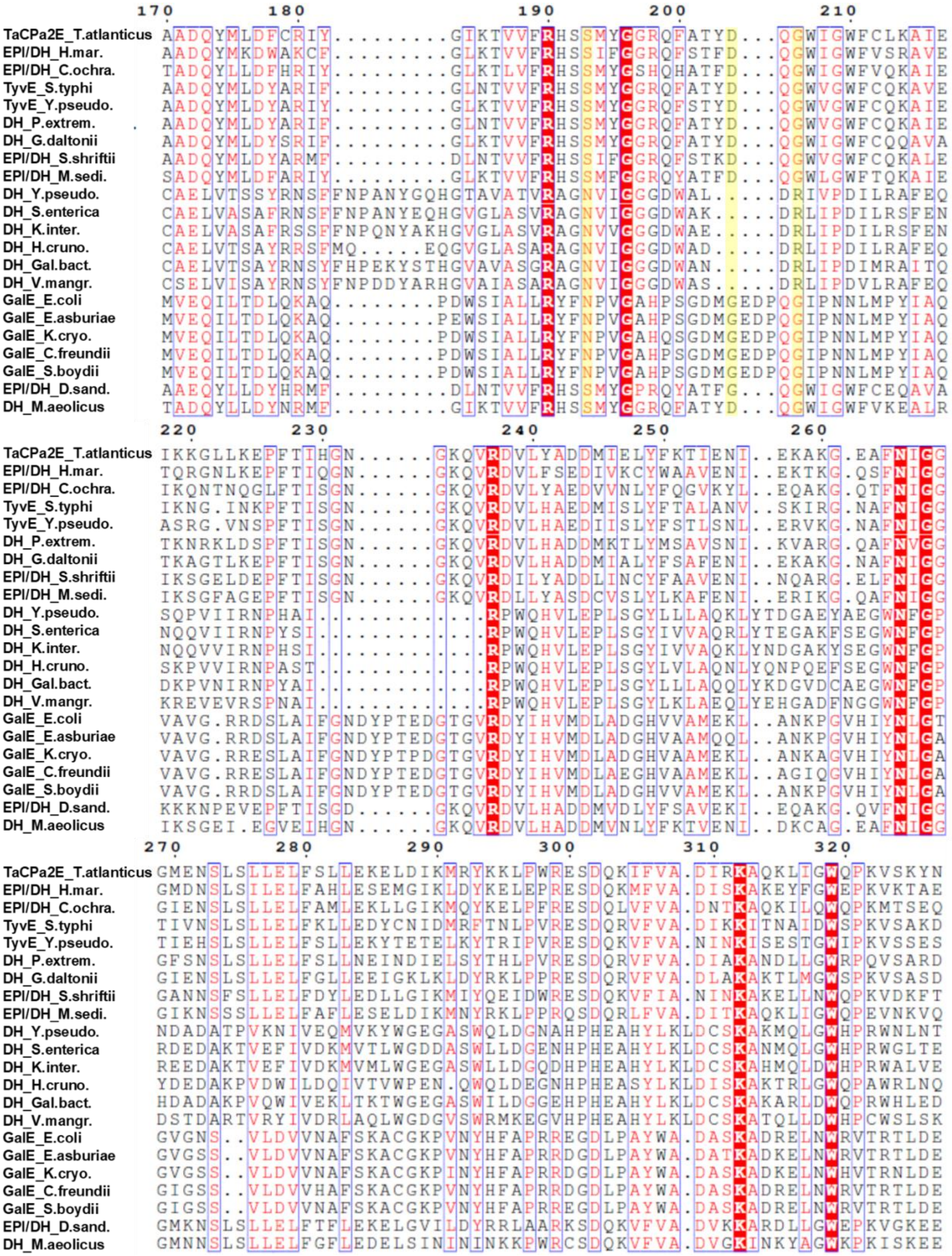
170 180 190 200 210
TaCPa2E_T.atlanticus AADQYMLDFCRIY.........GIKTVVFRHSSMYGGRQFATYD...QGWIGWFCLKAIE
EPI/DH_H.mar. AADQYMKDWAKCF.........GLKTVVFRHSSIFGGRQFSTFD...QGWIGWFVSRAVE
EPI/DH_C.ochra. TADQYLLDFHRIY.........GLKTLVFRHSSMYGSHQHATFD...QGWIGWFVQKAIE
TyvE_S.typhi AADQYMLDYARIF.........GLNTVVFRHSSMYGGRQFATYD...QGWVGWFCQKAVE
TyvE_Y.pseudo. AADQYMLDYARIY.........GLKTVVFRHSSMYGGRQFSTYD...QGWVGWFCQKAIE
DH_P.extrem. AADQYMLDYARIF.........GLNTVVFRHSSMYGGRQFATYD...QGWVGWFCQKAIE
DH_G.daltonii AADQYMLDYSRIF.........GLKTVVFRHSSMYGGRQFATYD...QGWIGWFCQQAVA
EPI/DH_S.shriftii AADQYMLDYARMF.........DLNTVVFRHSSIFGGRQFSTKD...QGWVGWFCQKALE
EPI/DH_M.sedi. SADQYMLDFARIY.........GLKTVVFRHSSMFGGRQYATFD...QGWLGWFTQKAIE
DH_Y.pseudo. CAELVTSSYRNSFFNPANYGQHGTAVATVRAGNVIGGGDWAL.....DRIVPDILRAFEQ
DH_S.enterica CAELVASAFRNSFFNPANYEQHGVGLASVRAGNVIGGGDWAK.....DRLIPDILRSFEN
DH_K.inter. CAELVASAFRSSFFNPQNYAKHGVGLASVRAGNVVGGGDWAE.....DRLIPDILRSFEN
DH_H.cruno. CAELVTSAYRRSFMQ.....EQGVGLASARAGNVIGGGDWAD.....DRLIPDILRAFEQ
DH_Gal.bact. CAELVTSAYRNSYFHPEKYSTHGVAVASGRAGNVIGGGDWAN.....DRLIPDIMRAITQ
DH_V.mangr. CSELVISAYRNSYFNPDDYARHGVAIASARAGNVIGGGDWAS.....DRLIPDVLRAFEQ
GalE_E.coli MVEQILTDLQKAQ........PDWSIALLRYFNPVGAHPSGDMGEDPQGIPNNLMPYIAQ
GalE_E.asburiae MVEQILTDLQKAQ........PEWSIALLRYFNPVGAHPSGDMGEDPQGIPNNLMPYIAQ
GalE_K.cryo. MVEQILTDLQKAQ........PDWSIALLRYFNPVGAHQSGDMGEDPQGIPNNLMPYIAQ
GalE_C.freundii MVEQILTDLQKAQ........PDWSIALLRYFNPVGAHPSGDMGEDPQGIPNNLMPYIAQ
GalE_S.boydii MVEQILTDLQKAQ........PDWSIALLRYFNPVGAHPSGDMGEDPQGIPNNLMPYIAQ
EPI/DH_D.sand. AAEQYLLDYHRMF.........DLNTVVFRHSSMYGPRQYATFG...QGWIGWFCEQAVA
DH_M.aeolicus TADQYLLDYNRMF.........GIKTVVFRHSSMYGGRQFATYD...QGWIGWFVKEALR
220 230 240 250 260
TaCPa2E_T.atlanticus IKKGLLKEPFTIHGN......GKQVRDVLYADDMIELYFKTIENI..EKAKG.EAFNIGG
EPI/DH_H.mar. TQRGNLKEPFTIQGN......GKQVRDVLFSEDIVKCYWAAVENI..EKTKG.QSFNIGG
EPI/DH_C.ochra. IKQNTNQGLFTISGN......GKQVRDVLYAEDVVNLYFQGVKYL..EQAKG.QTFNIGG
TyvE_S.typhi IKNG.INKPFTISGN......GKQVRDVLHAEDMISLYFTALANV..SKIRG.NAFNIGG
TyvE_Y.pseudo. ASRG.VNSPFTISGN......GKQVRDVLHAEDIISLYFSTLSNL..ERVKG.NAFNIGG
DH_P.extrem. TKNRKLDSPFTISGN......GKQVRDVLHADDMKTLYMSAVSNI..KVARG.QAFNVGG
DH_G.daltonii TKAGTLKEPFTISGN......GKQVRDVLHADDMIALYFSAFENI..EKAKG.NAFNIGG
EPI/DH_S.shriftii IKSGELDEPFTISGN......GKQVRDILYADDLINCYFAAVENI..NQARG.ELFNIGG
EPI/DH_M.sedi. IKSGFAGEPFTISGN......GKQVRDLLYASDCVSLYLKAFENI..ERIKG.QAFNIGG
DH_Y.pseudo. SQPVIIRNPHAI.............RPWQHVLEPLSGYLLLAQKLYTDGAEYAEGWNFGP
DH_S.enterica NQQVIIRNPYSI.............RPWQHVLEPLSGYIVVAQRLYTEGAKFSEGWNFGP
DH_K.inter. NQQVVIRNPHSI.............RPWQHVLEPLSGYIVVAQKLYNDGAKYSEGWNFGP
DH_H.cruno. SKPVVIRNPAST.............RPWQHVLEPLSGYLVLAQNLYQNPQEFSEGWNFGP
DH_Gal.bact. DKPVNIRNPYAI.............RPWQHVLEPLSGYLLLAQQLYKDGVDCAEGWNFGP
DH_V.mangr. KREVEVRSPNAI.............RPWQHVLEPLSGYLKLAEQLYEHGADFNGGWNFGP
GalE_E.coli VAVG.RRDSLAIFGNDYPTEDGTGVRDYIHVMDLADGHVVAMEKL..ANKPGVHIYNLGT
GalE_E.asburiae VAVG.RRDSLAIFGNDYPTEDGTGVRDYIHVMDLADGHVAAMQQL..ANKPGVHIYNLGA
GalE_K.cryo. VAVG.RRESLAIFGNDYPTPDGTGVRDYIHVMDLADGHVAAMEKL..ANKAGVHIYNLGA
GalE_C.freundii VAVG.RRESLAIFGNDYPTEDGTGVRDYIHVMDLAEGHVAAMEKL..AGIQGVHIYNLGA
GalE_S.boydii VAVG.RRDSLAIFGNDYPTEDGTGVRDYIHVMDLADGHVVAMEKL..ANKPGVHIYNLGA
EPI/DH_D.sand. KKKNPEVEPFTISGD......GKQVRDVLHADDMVDLYFSAVEKI..EQAKG.QVFNIGG
DH_M.aeolicus IKSGEI.EGVEIHGN......GKQVRDVLHADDMVNLYFKTVENI..DKCAG.EAFNIGG
270 280 290 300 310 320
TaCPa2E_T.atlanticus GMENSLSLLELFSLLEKELDIKMRYKKLPWRESDQKIFVA.DIRKAQKLIGWQPKVSKYN
EPI/DH_H.mar. GMDNSLSILELFAHLESEMGIKLDYKELEPRESDQKMFVA.DISKAKEYFGWEPKVKTAE
EPI/DH_C.ochra. GIENSLSLLELFAMLEKLLGIKMQYKELPFRESDQLVFVA.DNTKAQKILQWQPKMTSEQ
TyvE_S.typhi TIVNSLSLLELFKLLEDYCNIDMRFTNLPVRESDQRVFVA.DIKKITNAIDWSPKVSAKD
TyvE_Y.pseudo. TIEHSLSLLELFSLLEKYTETELKYTRIPVRESDQKVFVA.NINKISESTGWIPKVSSES
DH_P.extrem. GFSNSLSLLELFSLLNEINDIELSYTHLPVRESDQRVFVA.DIAKANDLLGWRPQVSARD
DH_G.daltonii GIENSLSLLELFGLLEEIGKLKLDYRKLPPRESDQRVFVA.DLAKAKTLMGWSPKVSASD
EPI/DH_S.shriftii GANNSFSLLELFDYLEDLLGIKMIYQEIDWRESDQKVFIA.NINKAKELLNWQPKVDKFT
EPI/DH_M.sedi. GIKNSSSLLELFAFLESELDIKMNYRKLPPRQSDQRLFVA.DITKAQKLIGWQPEVNKVQ
DH_Y.pseudo. NDADATPVKNIVEQMVKYWGEGASWQLDGNAHPHEAHYLKLDCSKAKMQLGWHPRWNLNT
DH_S.enterica RDEDAKTVEFIVDKMVTLWGDDASWLLDGENHPHEAHYLKLDCSKANMQLGWHPRWGLTE
DH_K.inter. REEDAKTVEFIVDKMVMLWGEGASWLLDGQDHPHEAHYLKLDCSKAHMQLDWHPRWALVE
DH_H.cruno. YDEDAKPVDWILDQIVTVWPEN.QWQLDEGNHPHEASYLKLDISKAKTRLGWQPAWRLNQ
DH_Gal.bact. HDADAKPVQWIVEKLTKTWGEGASWILDGGEHPHEAHYLKLDCSKAKARLDWQPRWHLED
DH_V.mangr. DSTDARTVRYIVDRLAQLWGDGVSWRMKEGVHPHEAHYLKLDCSKATQLLDWHPCWSLSK
GalE_E.coli GVGNS..VLDVVNAFSKACGKPVNYHFAPRREGDLPAYWA.DASKADRELNWRVTRTLDE
GalE_E.asburiae GVGSS..VLDVVNAFSKACGKPVNYHFAPRRDGDLPAYWA.DATKADKELNWRVTRTLDE
GalE_K.cryo. GVGSS..VLDVVNAFSKACGKPINYHFAPRRDGDLPAYWA.DASKADKELNWHVTRNLDE
GalE_C.freundii GIGSS..VLDVVHAFSKACGKPVNYHFAPRRDGDLPAYWA.DASKADRELNWRVTRTLDE
GalE_S.boydii GIGSS..VLDVVNAFSKACGKPVNYHFAPRREGDLPAYWA.DASKADRELNWRVTRTLDE
EPI/DH_D.sand. GMKNSLSLLELFTFLEKELGVILDYRRLAARKSDQKVFVA.DVKKARDLLGWEPKVGKEE
DH_M.aeolicus GMNNSLSLLELFGFLEDELSININKKPWRCSDQKVFVA.DVGKINKYAGWKPKISKEE

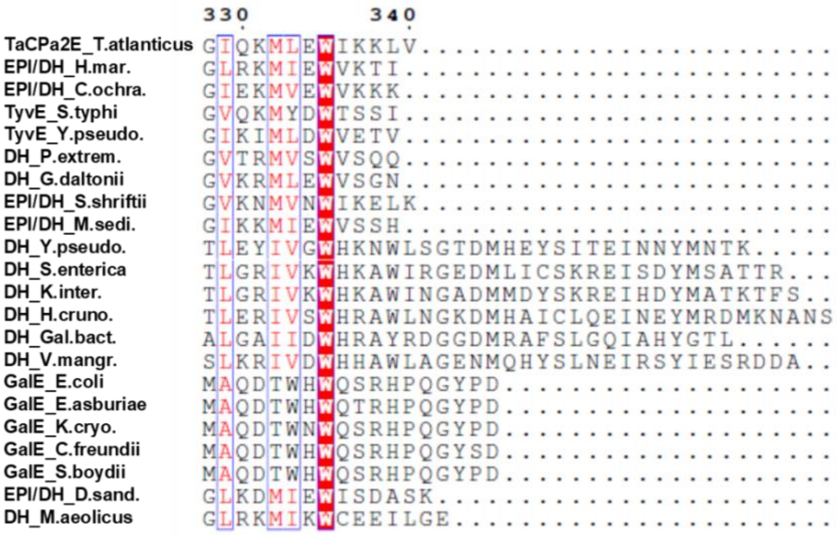

FIG S1 Multiple sequence alignment of selected putative and characterized TyvEs, GalEs and CDP-D-glucose-4,6-dehydratases. Highly conserved residues are highlighted in red. Yellow-labelled residues correspond to model-derived assumptions on substrate recognition in the enzyme's active site. Epimerase/dehydratase (TaCPa2E_T.atlanticus; from *Thermodesulfatator atlanticus;* NCBI: WP_022854415.1); Epimerase/dehydratase (EPI/DH_H.mar.; from *Halobacteriovorax marinus;* NCBI: WP_096908434.1); Epimerase/dehydratase (EPI/DH_C.ochra.; from *Capnocytophaga ochracea;* NCBI: WP_015781970.1); Epimerase (TyvE_S.typhi; from *Salmonella typhi;* NCBI: WP_000770936); Epimerase (TyvE_Y.pseudo.; from *Yersinia pseudotuberculosis;* NCBI: AKA20962.1); CDP-D-glucose 4,6-dehydratase (DH_P.extrem.; from *Pseudomonas extremaustralis;* NCBI: WP_010566515.1); CDP-D-glucose 4,6-dehydratase (DH_G.daltonii; from *Geobacter daltonii;* NCBI: WP_012647211.1); Epimerase/dehydratase (EPI/DH_S.shriftii; from *Selenihalanaerobacter shriftii;* NCBI: WP_078811136.1); Epimerase/dehydratase (EPI/DH_M.sedi.; from *Mangrovitalea sediminis;* NCBI: WP_097460147.1); CDP-D-glucose 4,6-dehydratase (DH_Y.pseudo.; from *Yersinia pseudotuberculosis;* NCBI: WP_072080415.1); CDP-D-glucose 4,6-dehydratase (DH_S.enterica; from *Salmonella enterica;* NCBI: EAA4947084.1); CDP-D-glucose 4,6-dehydratase

(DH_K.inter.; from *Kluyvera intermedia;* NCBI: WP_062778321.1); CDP-D-glucose 4,6-dehydratase (DH_H.cruno.; from *Hydrogenovibrio crunogenus;* NCBI: WP_062778321.1); CDP-D-glucose 4,6-dehydratase (DH_Gal.bact.; from *Gallionellaceae bacterium;* NCBI: TAJ82294.1); CDP-D-glucose 4,6-dehydratase (DH_V.mangr.; from *Vibrio mangrovi;* NCBI: WP_087481551.1); UDP-D-glucose 4-epimerase (GalE_E.coli; from *Escherichia coli;* NCBI: 1LRJ_A); UDP-D-glucose 4-epimerase (GalE_E.asburiae; from *Enterobacter asburiae;* NCBI: WP_057060194.1); UDP-D-glucose 4-epimerase (GalE_K.cryo.; from *Kluyvera cryocrescens;* NCBI: WP_061282664.1); UDP-D-glucose 4-epimerase (GalE_C.freundii; from *Citrobacter freundii;* NCBI: WP_086503853.1); UDP-D-glucose 4-epimerase (GalE_S.boydii; from *Shigella boydii;* NCBI: WP_073817003.1); Epimerase/dehydratase (EPI/DH_D.sand.; from *Dethiosulfatarculus sandiegensis;* NCBI: WP_044352701.1); CDP-D-glucose 4,6-dehydratase (DH_M.aeolicus; from *Methanococcus aeolicus;* NCBI: WP_011973107.1).

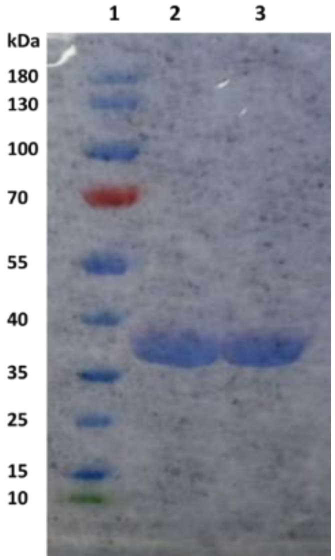

FIG S2 Results of SDS-PAGE of purified *Ta*CPa2E (~39.3 kDa). Lane 1: molecular mass ladder, lane 2 and 3: concentrated protein samples (25 μg).

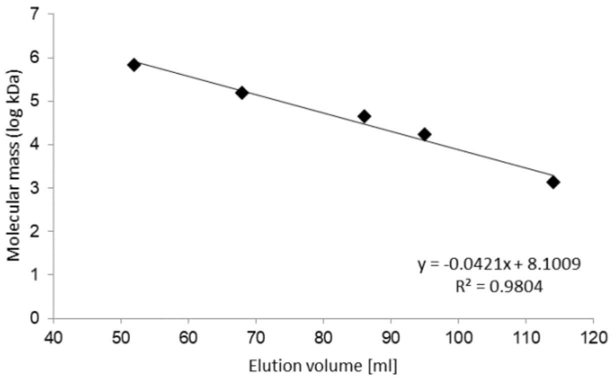

FIG S3 Gel filtration standard curve: The column (HiLoad 16/60 Superdex 200) was calibrated with gel filtration standard mixture #1511901. Logarithmic molecular masses of thyroglobulin (670000 Da), bovine γ-globulin (158000 Da), chicken ovalbumin (44000 Da), equine myoglobin (17000 Da) and vitamin B12 (1350 Da) are plotted against their respective elution volumes. A flow rate of 1 mL/min was applied. Buffer: 10 mM HEPES, 150 mM NaCl, 5% glycerol at pH 7. The protein standard curve (BioRad molecular mass standards) was used to determine the molecular mass of HisTag-purified *Ta*CPa2E.

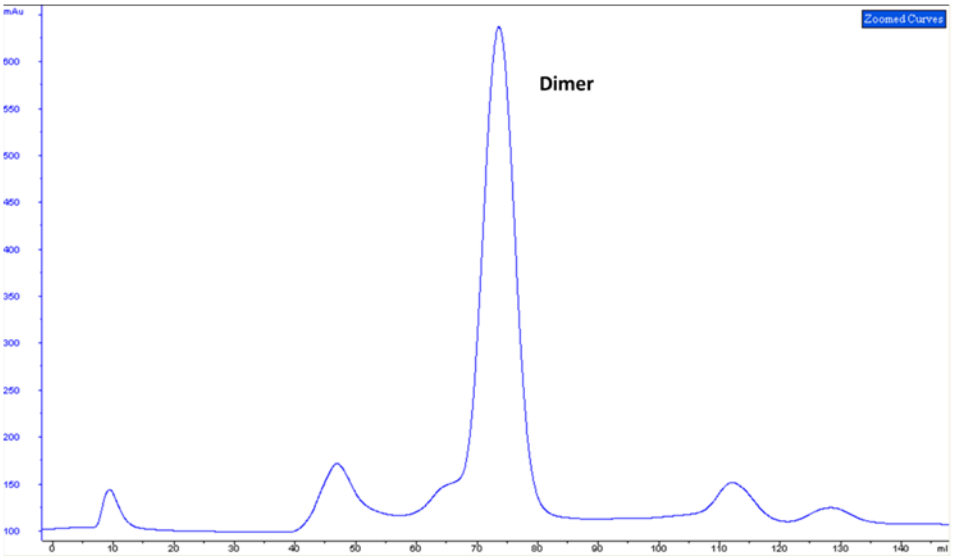

FIG S4 Gel filtration chromatogram of *Ta*CPa2E at 280 nm UV-absorption. The apparent molecular mass of the dimer with an elution volume of 75 ml is labelled. The sample volume applied was 2 mL containing 1.52 mM *Ta*CPa2E (60 mg/mL).

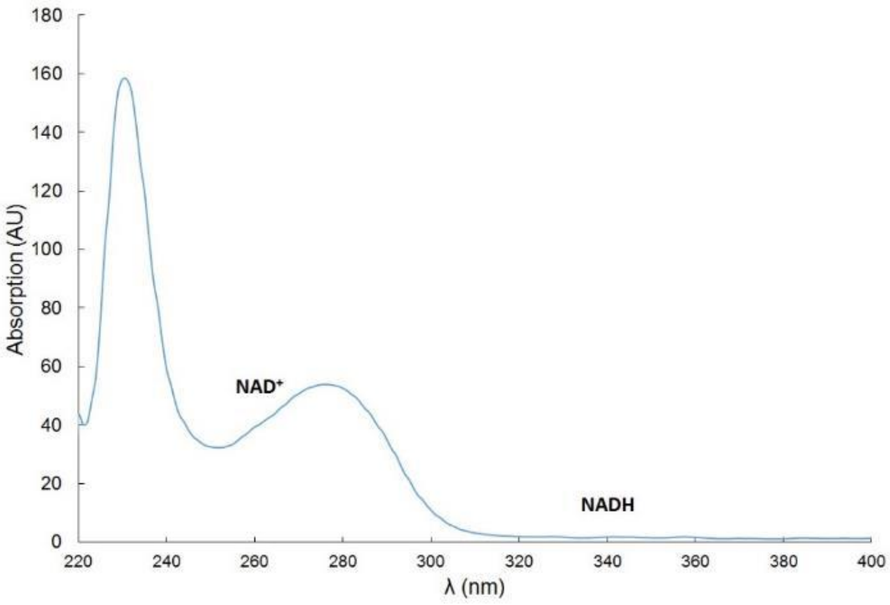

FIG S5 Absorbance spectrum of *Ta*CPa2E (762 µM, 30 mg/mL) showing the presence of NAD^+. Peaks of NAD^+ (260 nm) and less pronounced NADH (340 nm) are indicated.

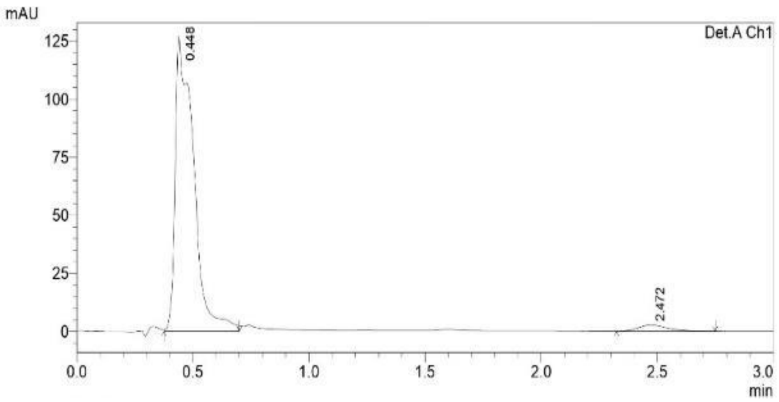

FIG S6 Analytical HPLC chromatogram of NAD^+ and NADH extracted from *Ta*CPa2E. NAD^+ (retention time: 0.448 min) and NADH (retention time: 2.472 min) were identified using authentic standards. Cofactors were eluted applying an isocratic flow (2 mL/min) at 40 °C with a mobile phase composed of 95% A: 20 mM potassium phosphate buffer, 40 mM tetrabutylammonium bromide (pH 5.9) and 5% B: acetonitrile. Column: 50 × 4.6 mm, 5 µm C18 100 Å from Phenomenex, Germany.

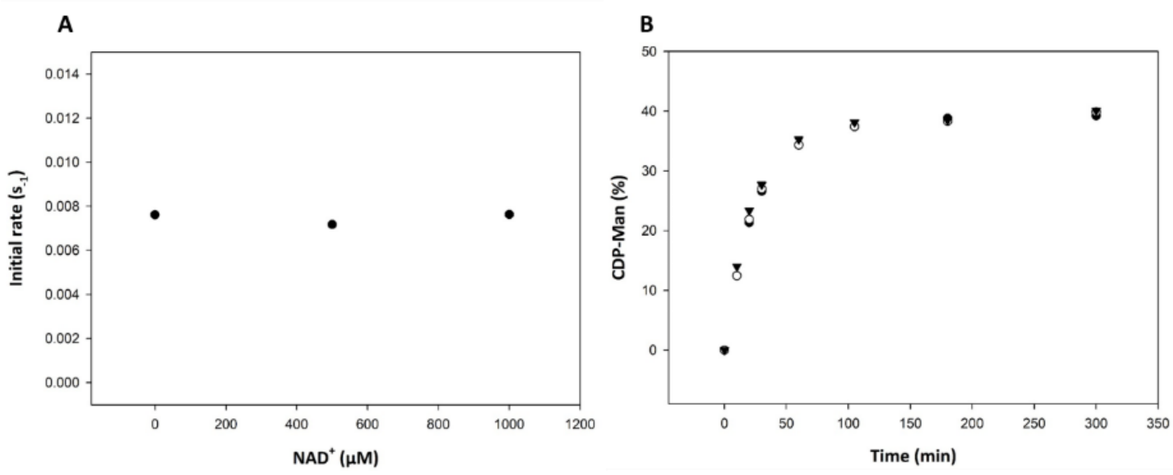

FIG S7 Effect of NAD^+ on the activity and the equilibrium of *Ta*CPa2E with CDP-Glc. **A.** Dependency of initial rates (s^{-1}) on NAD^+ concentrations (0, 500 and 1000 µM). **B.** Corresponding time courses of CDP-Glc conversions: Black circles (0 mM NAD^+), white circles (0.5 mM NAD^+) and triangles (1 mM NAD^+). Reactions were carried out with 1 mM CDP-Glc and 25.4 µM *Ta*CPa2E (1 mg/ml) in 100 mM MOPS buffer (pH 7.5) at 60 °C.

Sugar nucleotide synthesis I

CDP-α-D-glucose

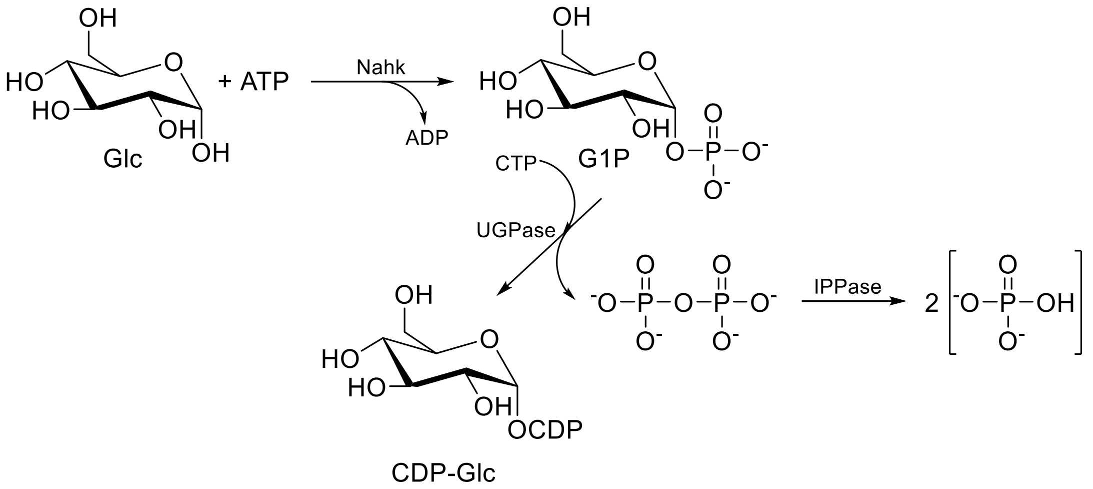

Scheme S1 Enzymatic synthesis route of CDP-α-D-glucose (CDP-Glc) starting from D-glucose. The first step comprises phosphorylation of the sugar by an anomeric kinase producing glucose-1-phosphate (G1P). The reaction mixture (15 mL) contained 5 mg/mL N-acetylhexosamine 1-kinase (NahK), 20 mM ATP, 5 mM $MgCl_2$ and 25 mM D-glucose in MOPS buffer (100 mM, pH 7.5). Incubation at 30 °C was carried out for 16 h until ATP was fully depleted. Phosphorylation progress was monitored with TLC. The second step involves the nucleotidyl transfer from cytidine-5'-triphosphate (CTP) to G1P. The reaction was catalyzed by 1 mg/mL UDP-glucose pyrophosphorylase (UGPase) in the presence of 2 mg/mL inorganic pyrophosphatase (IPPase) and 25 mM CTP. The reaction time was 20 h.

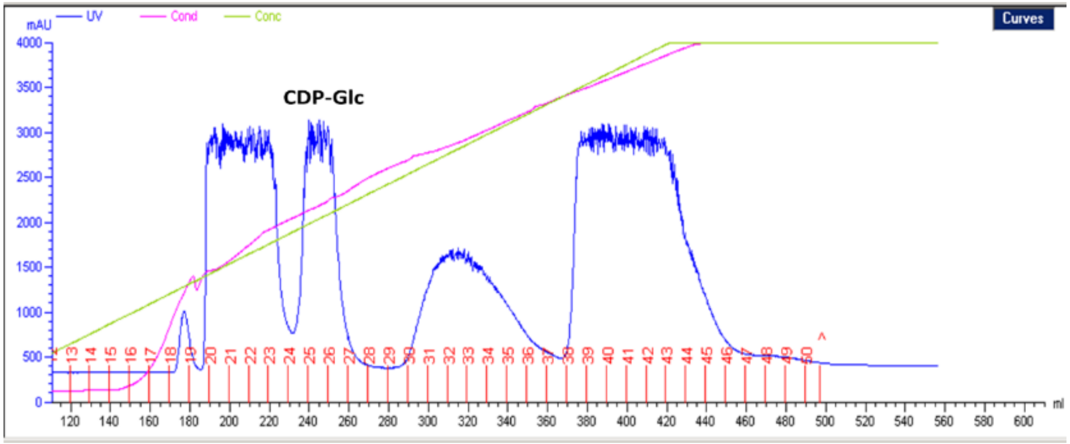

FIG S8 An Äkta chromatogram of preparative anion exchange chromatographic purification of CDP-Glc (labelled). The blue line corresponds to the UV signal at 280 nm. The light green line shows the linear gradient (100% after 4 h) used for eluting the products. A flow rate of 2 mL/min and a sample volume of 10 mL (~10 mM CDP-Glc) were applied.

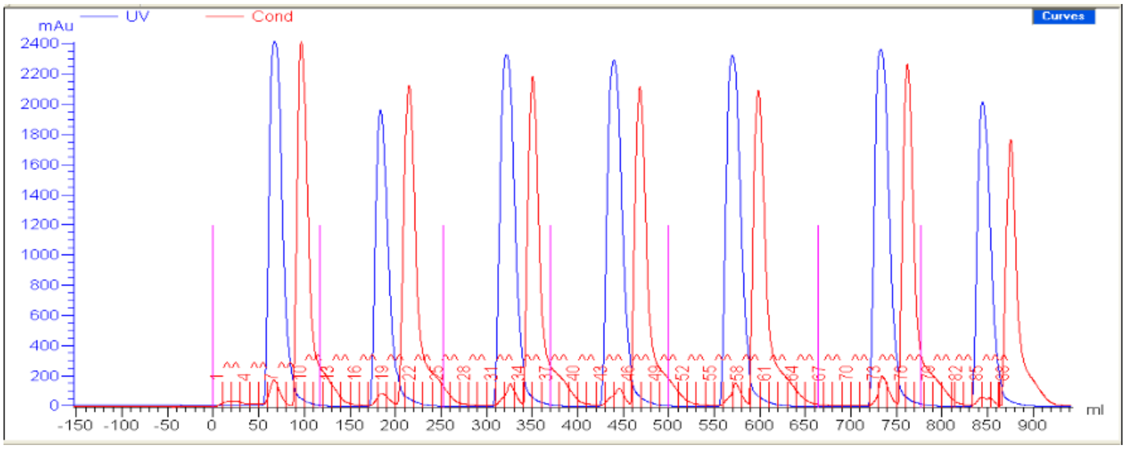

FIG S9 An Äkta chromatogram of preparative size-exclusion chromatographic purification of CDP-Glc. The blue line shows the UV absorbance of CDP-Glc at 280 nm and the red line corresponds to the conductivity signal of acetate.

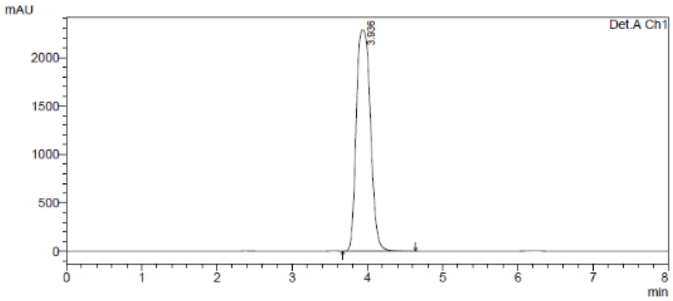

FIG S10 Analytical HPLC chromatogram of isolated CDP-Glc. The desired compound showed excellent UV purity.

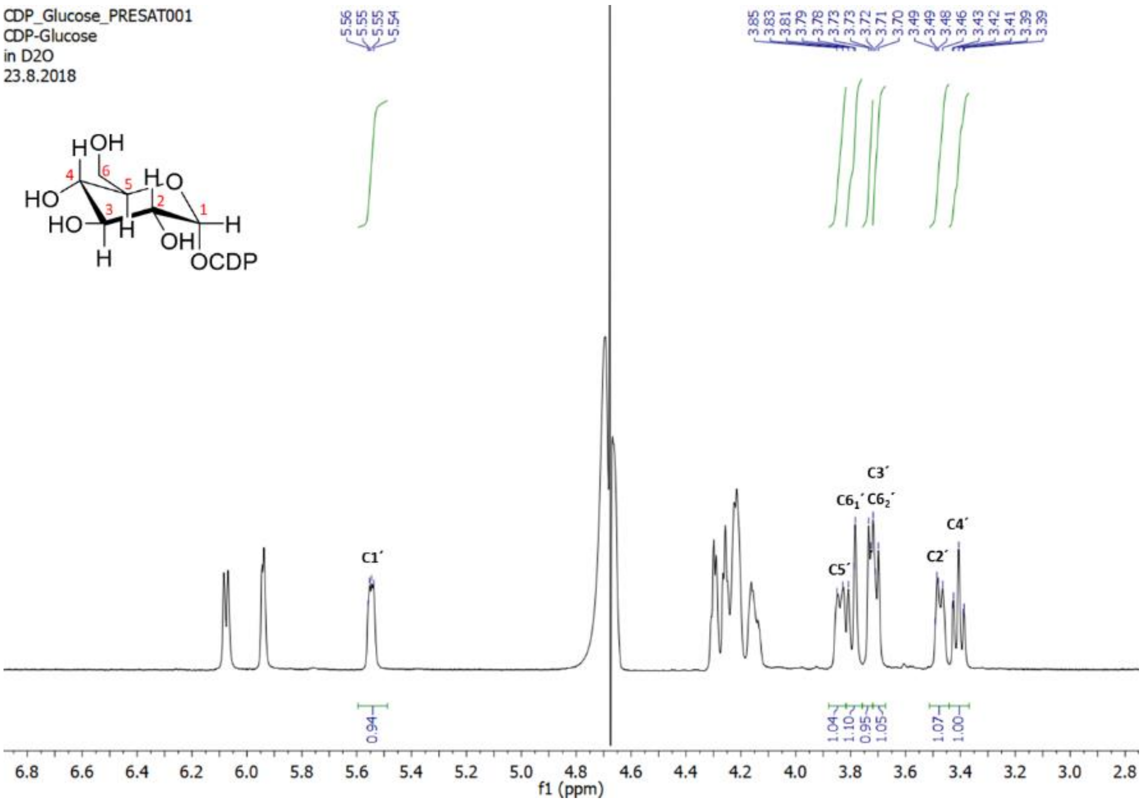

FIG S11 ^{1}H-NMR spectrum (500 MHz, D_2O) of CDP-Glc in 50 mM potassium phosphate buffer (pD 7.5). δ 5.55 ppm (dd, 1H), 3.85 ppm (m, 1H), 3.72 ppm (t, 1H), 3.71 ppm (d, 1H), 3.78 ppm (d, 1H), 3.48 ppm (dd, 1H), 3.40 ppm (t, 1H).

CDP-α-D-mannose

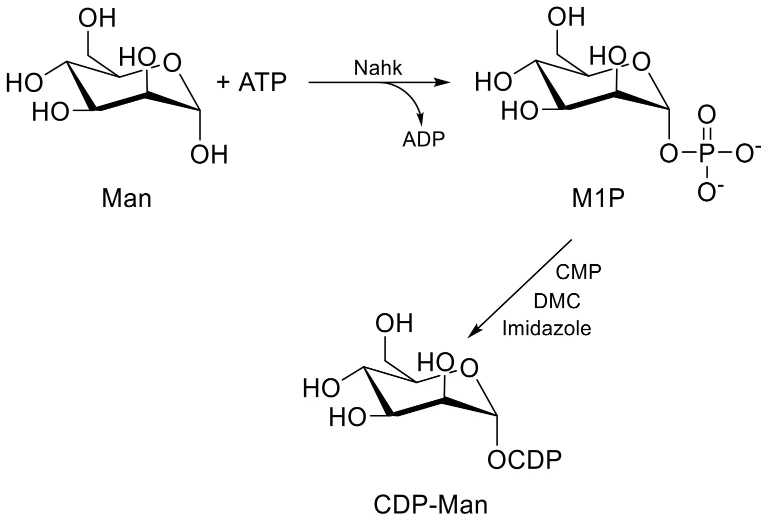

Scheme S2 Representation of the chemo-enzymatically synthesized CDP-D-mannose (CDP-Man). Phosphorylation of D-mannose to mannose-1-phosphate (M1P) was carried out using 5 mg/mL NahK, 20 mM ATP, 5 mM $MgCl_2$ and 25 mM D-mannose in 15 mL MOPS buffer (100 mM, pH 7.5). Incubation was performed at 30 °C for 16 h until full ATP depletion. The resulting D-mannose-1-phosphate was purified prior to chemical CMP-transfer by means of preparative anion exchange chromatography (Fig. S12). Chemical coupling of cytidine-5'-monophosphate (CMP) to M1P in the presence of imidazole and 2-chloro-1,3-dimethylimidazolinium chloride (DMC) was performed as described in published protocols (1). CDP-Man was pre-purified by preparative anion exchange (Fig. S13) and size-exclusion chromatography (Fig. S14). Remaining compounds (CMP-dimer, cytidine) were separated by preparative TLC (Fig. S15).

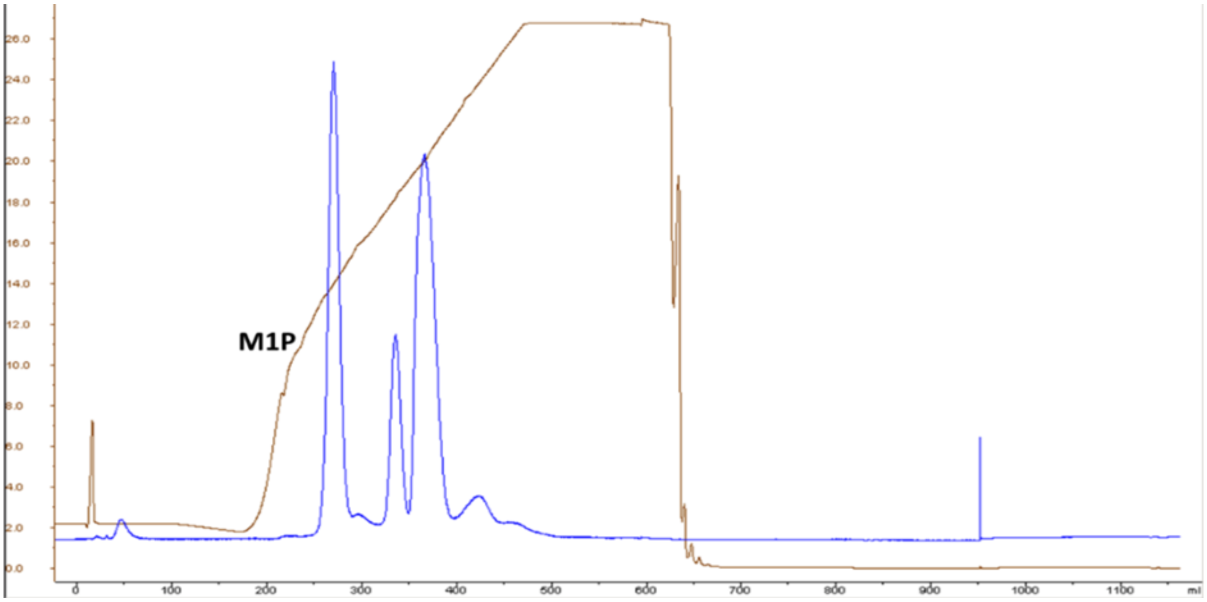

FIG S12 An Äkta chromatogram of preparative anion exchange chromatographic purification of mannose-1-phosphate (M1P). The blue line corresponds to the UV signal at 280 nm. The light brown line shows the conductivity used to detect the elution of M1P (10 – 12 mS). A flow rate of 2 mL/min with a sample volume of 10 mL (~10 mM M1P) and a 2h-linear gradient to 100% elution buffer were applied (binding buffer: 20 mM $NaHCO_3$; elution buffer: 300 mM $NaHCO_3$).

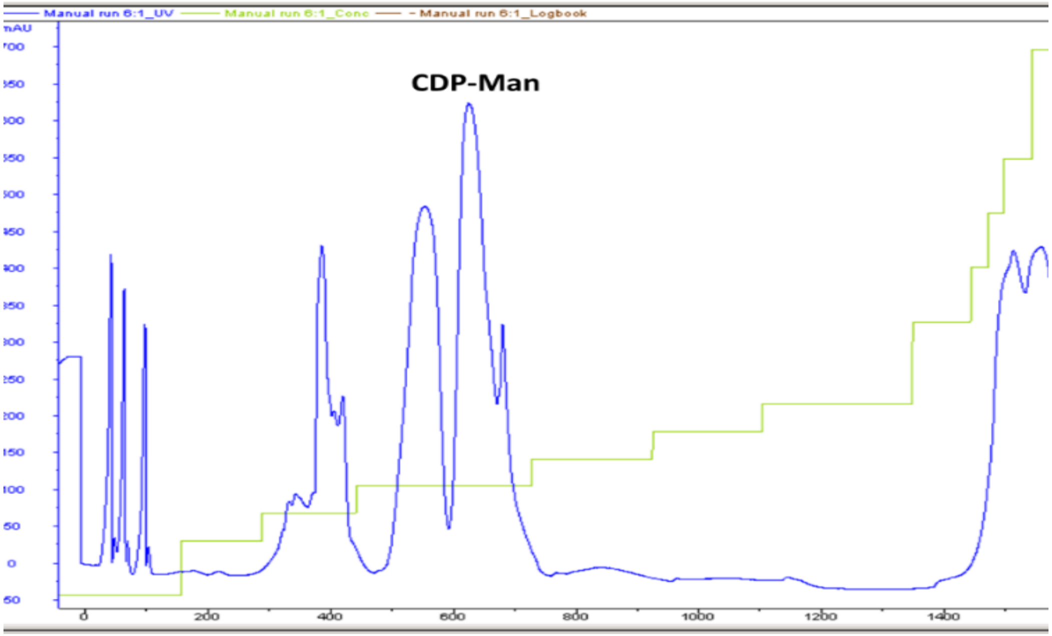

FIG S13 An Äkta chromatogram of preparative anion exchange chromatographic purification of CDP-Man (labelled). The blue line corresponds to the UV signal at 280 nm. The light green line shows the step-wise gradient of the buffer used for product elution. The steps were: 310 ml of 20 mM NaOAc, 130 ml of 100 mM NaOAc, 160 ml of 150 mM NaOAc, 290 ml of 200 mM NaOAc, 190 mL of 250 mM NaOAc, 180 ml of 300 mM NaOAc, 250 ml of 350 mM NaOAc, 100 ml of 500 mM NaOAc, 25 ml of 600 mM NaOAc, 25 ml of 700 mM NaOAc, 25 ml of 800 mM NaOAc, 50 ml of 1000 mM NaOAc, 50 ml of 500 mM NaOAc. A sample volume of 10 mL (~15 mM CMP) was used.

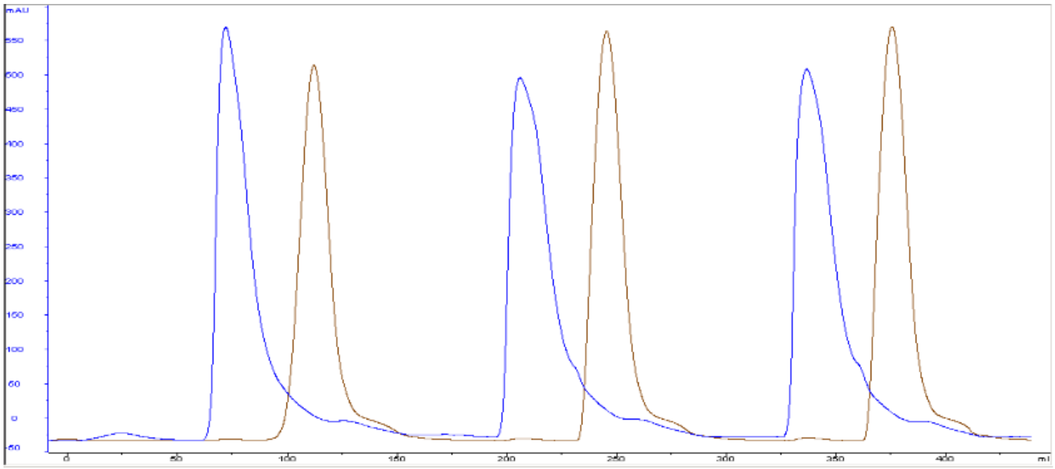

FIG S14 An Äkta chromatogram of preparative size-exclusion chromatographic purification of CDP-Man. The blue line corresponds to the UV absorbance of CDP-Man at 280 nm whereas the light brown line shows the conductivity signal of sodium acetate.

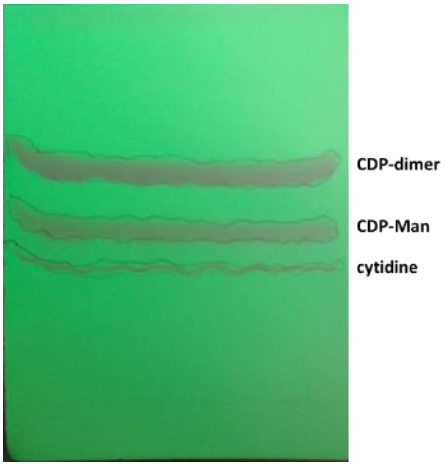

FIG S15 Silica plate of preparative thin-layer chromatographic purification of CDP-Man. An eluent containing isopropanol/ammonia (25%) in a ratio of 1/1 was used to separate the desired product.

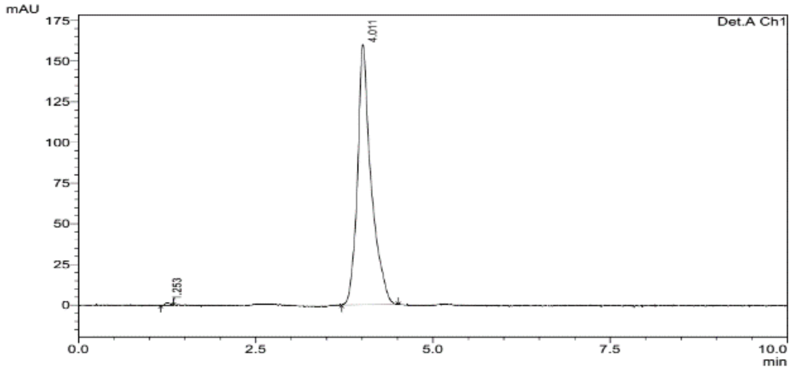

FIG S16 Analytical HPLC chromatogram of isolated CDP-Man. A purity of 99% (UV = 271 nm) was obtained.

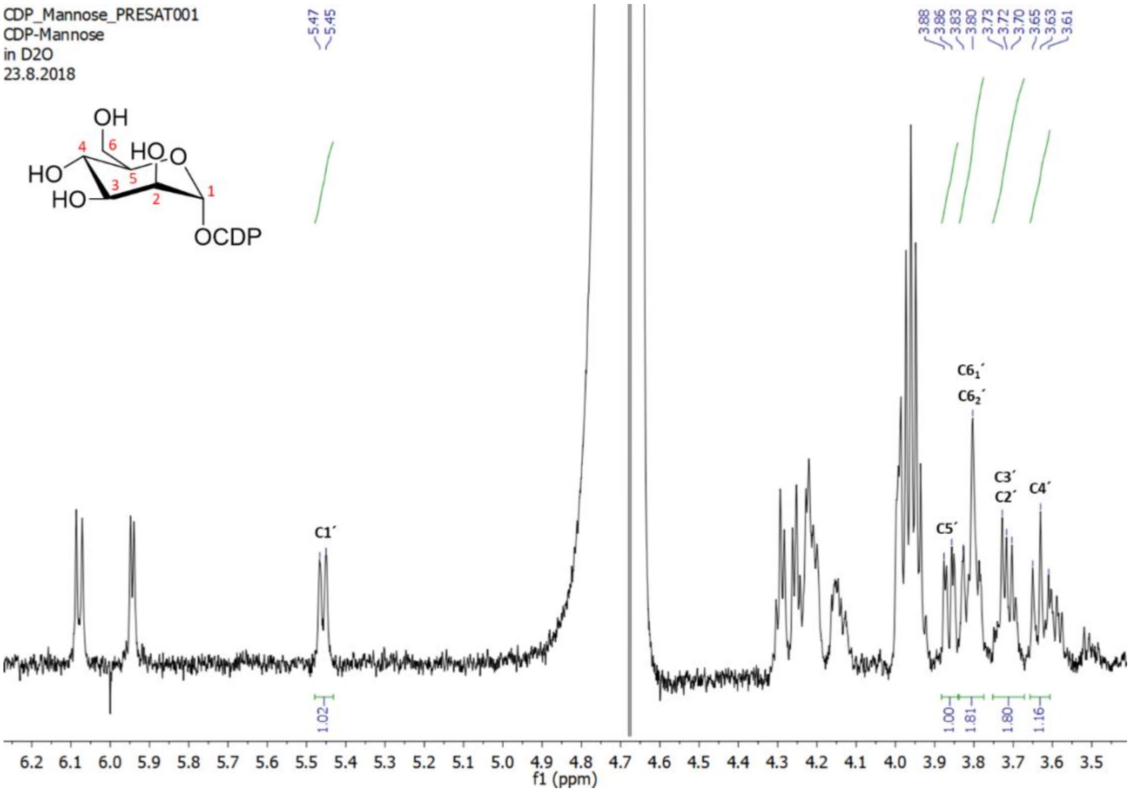

FIG S17 ^{1}H-NMR spectrum (500 MHz, D_2O) of CDP-Man in 50 mM potassium phosphate buffer (pD 7.5). δ 5.45 ppm (dd, 1H), 3.88 ppm (dd, 1H), 3.83 ppm (d, 1H), 3.80 ppm (m, 1H), 3.73 ppm (d, 1H), 3.70 ppm (dd, 1H), 3.63 ppm (t, 1H).

Sugar nucleotide synthesis II *CDP-α-D-glucose derivatives*

Enzymes for CDP-α-D-paratose synthesis

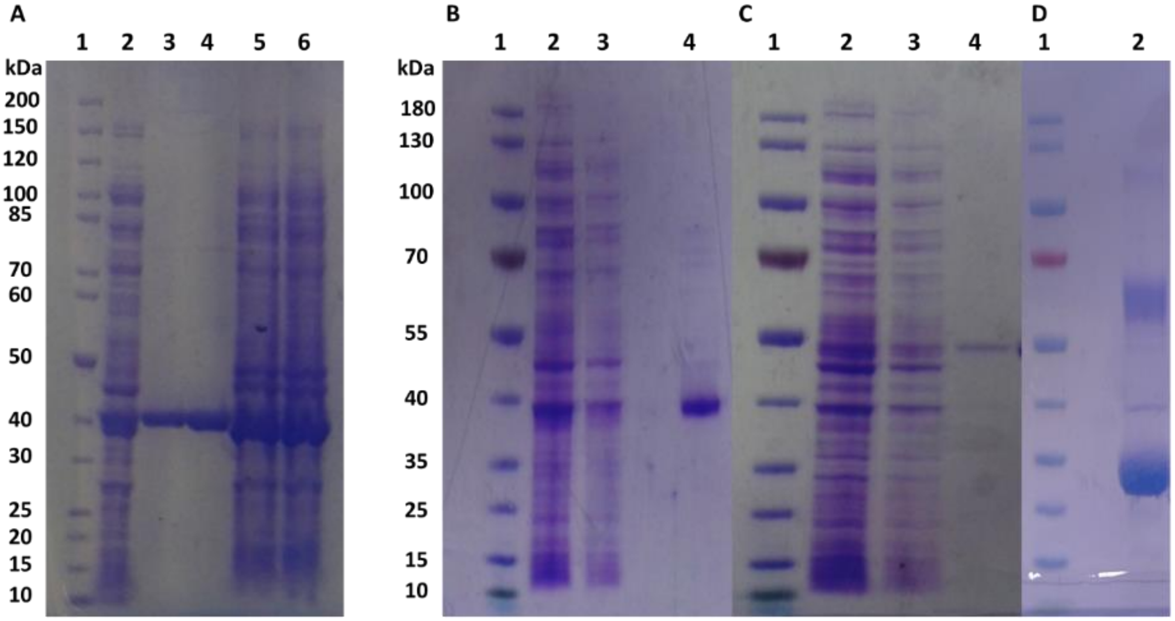

FIG S18 Results of SDS-PAGE of purified *S. typhi* CDP-D-glucose 4,6-dehydratase (**A**), *Y. pseudotuberculosis* E3 (**B**), *Y. pseudotuberculosis* E1 (**C**) and (**D**) *S. typhi* CDP-D-paratose synthase (mono- and dimeric) with a molecular mass of 41, 36, 48.3 and 31.5 kDa, respectively. 10-25 µg of protein were loaded. **A.** Lane 1: molecular mass ladder, lane 2: cell free extract, lanes 3, 4: concentrated protein sample, lanes 5, 6: lysate. **B.** Lane 1: molecular mass ladder, lanes 2, 3: lysate, lane 4: concentrated protein sample. **C.** Lane 1: molecular mass ladder, lanes 2, 3: lysate, lane 4: concentrated protein sample. **D.** Lane 1: molecular mass ladder, lane 2: concentrated protein sample.

CDP-6-deoxy-D-*xylo*-hexopyranos-4-ulose

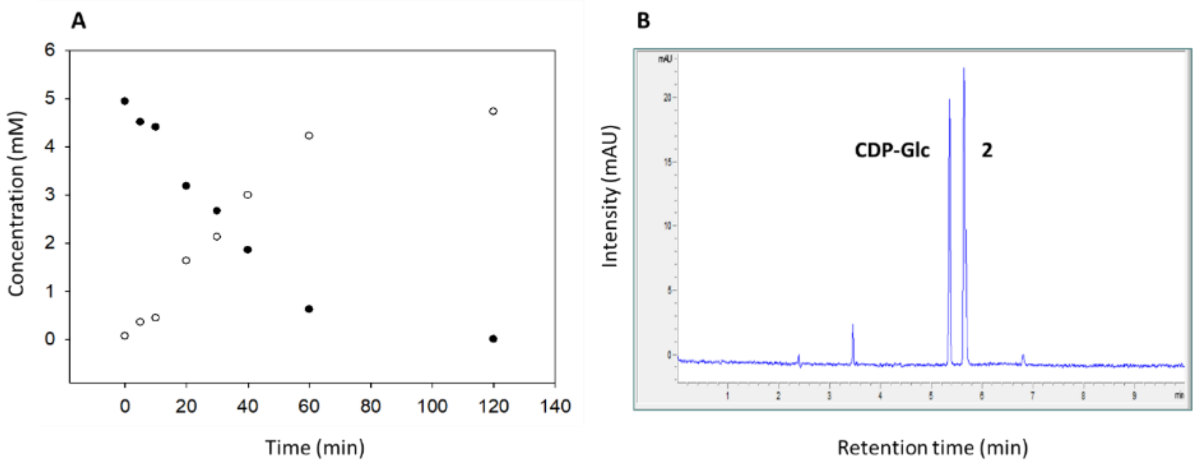

FIG S19 Determination of the specific activity of CDP-D-glucose 4,6-dehydratase with CDP-Glc as substrate. **A.** Time course of CDP-D-glucose 4,6-dehydratase reaction. Black filled circle: CDP-Glc concentration. White circle: Concentration of **2** (see Fig. 1). The activity was calculated from the slope of the linear fit to the initial rate period (0 - 60 min) of the curve, resulting in 3.68 U/mg. Conditions: 0.5 µM (0.02 mg/mL) enzyme, 5 mM CDP-Glc, 100 mM MOPS, 30 °C, pH 7.5. **B.** Capillary electrophoresis chromatogram of the conversion of CDP-Glc to **2** (see Fig. 1) catalyzed by CDP-D-glucose 4,6-dehydratase.

CDP-6-deoxy-α-D-glucose

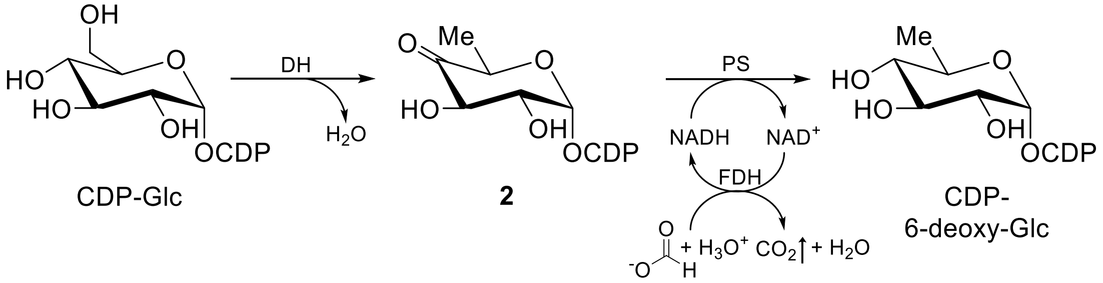

Scheme S3 Enzymatic synthesis route of CDP-6-deoxy-α-D-glucose (CDP-6-deoxy-Glc) starting from CDP-Glc. CDP-D-glucose 4,6-dehydratase (DH, 2 mg/mL) was used to catalyze the dehydration step of 20 mM CDP-Glc (10 mL reaction volume, 100 mM MOPS, 30 °C, pH 7.5). In the second step, 5 mg/mL CDP-D-paratose synthase, 2 mg/mL (40 U) formate dehydrogenase (FDH), 20 mM of **2** (see Fig. 1) and 0.2 mM NAD^+ in buffered ammonium formate (30 mM, pH 7.5) were added. Incubation was performed at 30 °C for 16 h. Formate dehydrogenase whole-cell catalyst was removed by centrifugation (5 min, 21130 g).

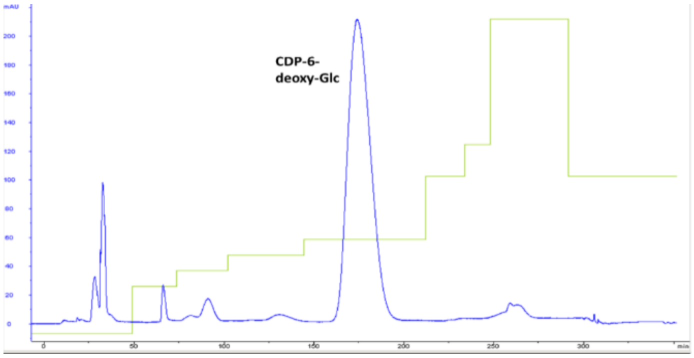

FIG S20 An Äkta chromatogram of preparative anion exchange chromatographic purification of CDP-6-deoxy-Glc (labelled). The blue line corresponds to the UV signal at 280 nm. The light green line shows the step-wise gradient of the elution buffer. The steps were: 200 ml of 20 mM NaOAc, 100 ml of 150 mM NaOAc, 100 ml of 200 mM NaOAc, 170 ml of 250 mM NaOAc, 270 mL of 300 mM NaOAc, 80 ml of 500 mM NaOAc, 70 ml of 60 mM NaOAc, 220 ml of

1000 mM NaOAc, 220 ml of 500 mM NaOAc. A sample volume of 10 mL (~20 mM CDP-6-deoxy-Glc) was applied.

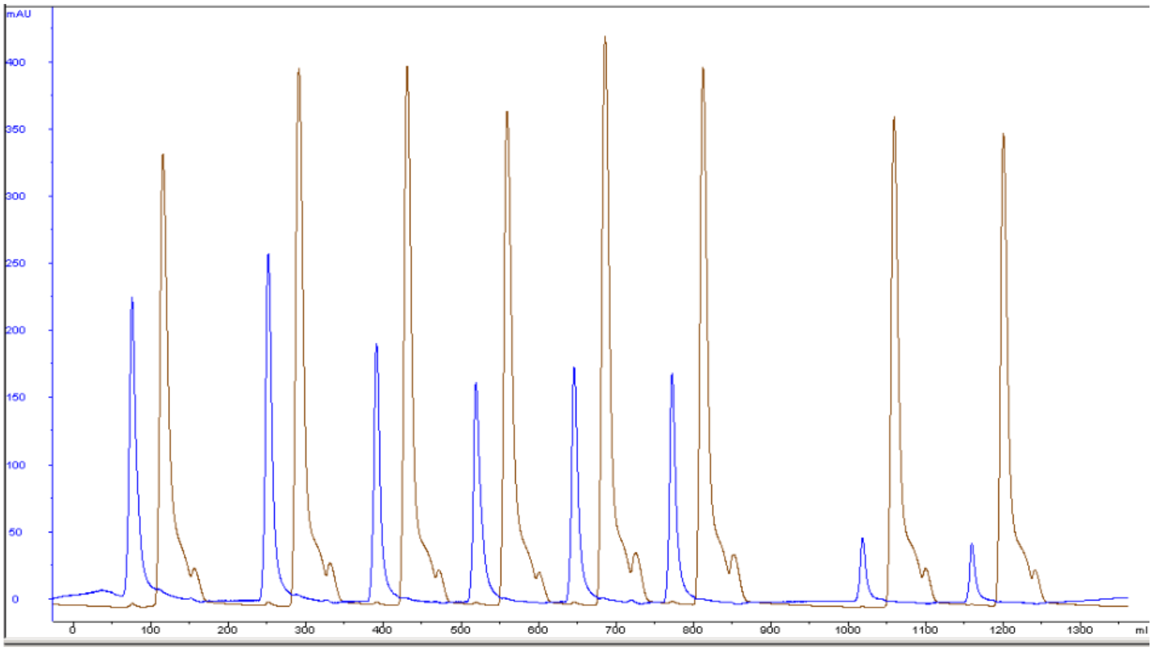

FIG S21 An Äkta chromatogram of preparative size-exclusion chromatographic purification of CDP-6-deoxy-Glc. The blue line corresponds to the UV absorbance of CDP-3-deoxy-Glc at 280 nm and the brown line shows the conductivity signal of acetate.

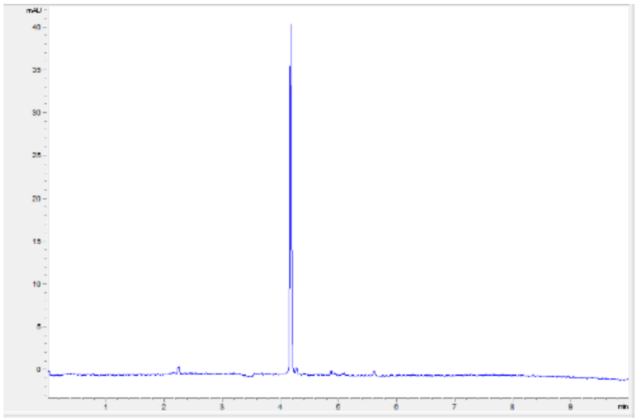

FIG S22 Capillary electrophoresis chromatogram of isolated CDP-6-deoxy-Glc. The desired product was detected at 271 nm UV absorption and obtained in excellent purity.

CDP-3,6-dideoxy-D-*xylo*-hexopyranos-4-ulose (attempted)

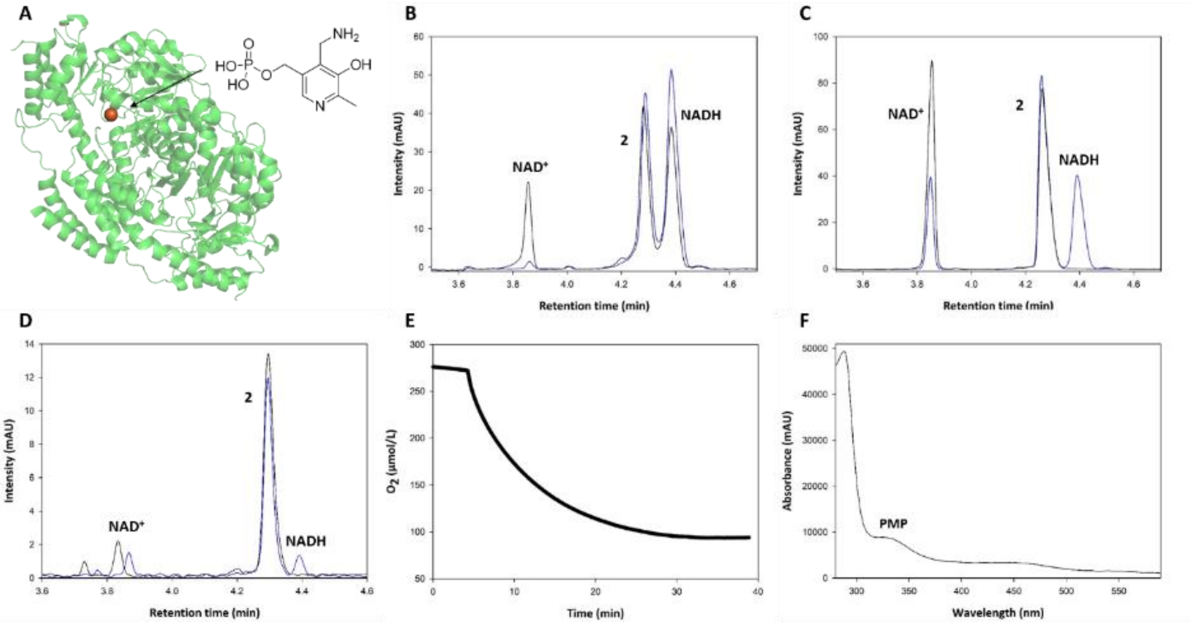

FIG S23 A. Crystal structure of E1 from *Y. pseudotuberculosis* (PDB: 3BCX) and pyridoxamine 5'-phosphate (PMP) acting as cofactor (2). Capillary electrophoresis chromatographic runs of reactions using (**B**) E1/E3, NADH and **2** (see Fig. 1), (**C**) E3 and NADH, clearly showing an NAD^+ increase, (**D**) anoxic conditions and an NADH-recycling system. Blue lines indicate reaction starting points, black lines advanced reaction time points. **E.** Oxygen consumption over time in the reaction of E3 with NADH forming H_2O_2 and NAD^+ as products (reduction of molecular oxygen coupled to oxidation of NADH). The specific activity of E3 was calculated from the linear part (5 – 10 min) of oxygen depletion and yielded 0.55 U/mg. **F.** Absorbance spectrum of E1 from *Y. pseudotuberculosis* with bound PMP indicated at 330 nm.

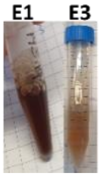

FIG S24 Brown color-indication for the presence of an Fe-S cluster in E1 and E3. Isolated and concentrated E1 (left), diluted E3 solution (right).

CDP-3-deoxy-α-D-glucose

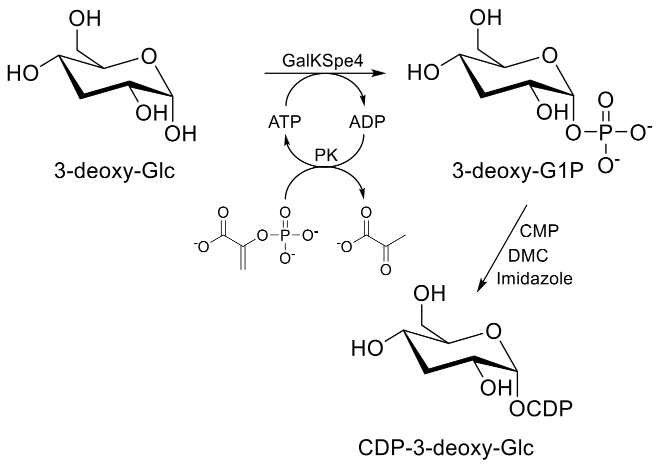

Scheme S4 Schematic representation of the chemo-enzymatic synthesis route of CDP-3-deoxy-α-D-glucose (CDP-3-deoxy-Glc). The first step comprises the phosphorylation of 3-deoxy-glucose by an anomeric kinase (GalKSpe4) coupled to an ATP-regeneration system using pyruvate kinase (PK) resulting in 3-deoxy-glucose-1-phosphate (3-deoxy-G1P) and pyruvate. The reaction mixture (15 mL) contained 2 mg/mL galactokinase (GalKSpe4), 40 U/mL pyruvate kinase (PK), 20 mM 3-deoxy-D-glucose, 5 mM $MgCl_2$, 60 mM phosphoenolpyruvate and 1 mM ATP in MOPS buffer (100 mM, pH 7.5). Incubation was carried out at 30 °C for 30 h to the point of complete substrate phosphorylation. Chemical coupling of cytidine-5'-monophosphate (CMP) to 3-deoxy-G1P catalyzed by imidazole and 2-chloro-1,3-dimethylimidazolinium chloride (DMC) was carried out following published protocols (1).

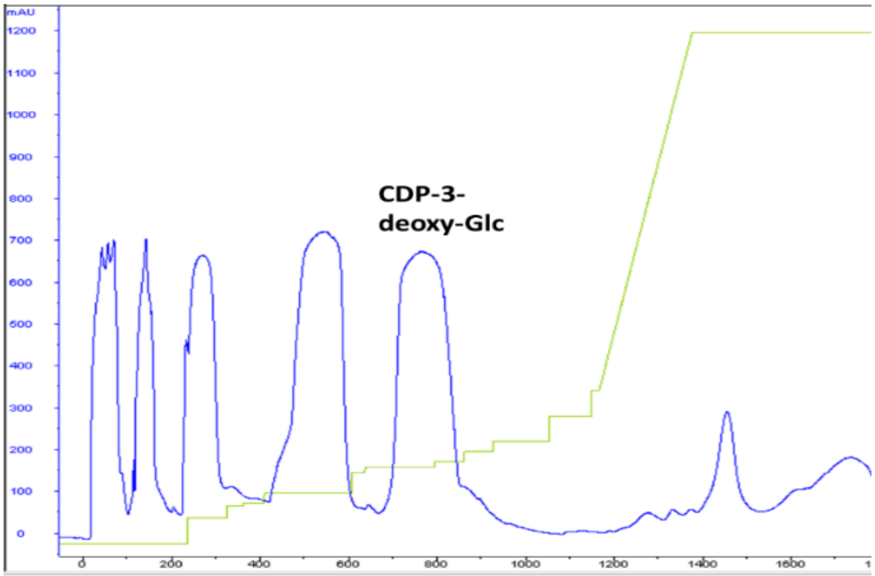

FIG S25 An Äkta chromatogram of preparative anion exchange chromatographic purification of CDP-3-deoxy-Glc (labelled). The blue line corresponds to the UV signal at 280 nm. The light green line shows the step-wise gradient of the elution buffer using an optimized protocol: The steps were: 230 ml of 20 mM NaOAc, 90 ml of 50 mM NaOAc, 40 ml of 75 mM NaOAc, 50 ml of 80 mM NaOAc, 200 mL of 100 mM NaOAc, 30 mL of 140 mM NaOAc, 50 mL of 150 mM NaOAc, 80 mL of 160 mM NaOAc, 115 mL of 200 mM NaOAc, 90 mL of 250 mM NaOAc, 20 mL of 300 mM NaOAc, 830 mL of 1000 mM NaOAc. A sample volume of 10 mL (~20 mM CMP) was used.

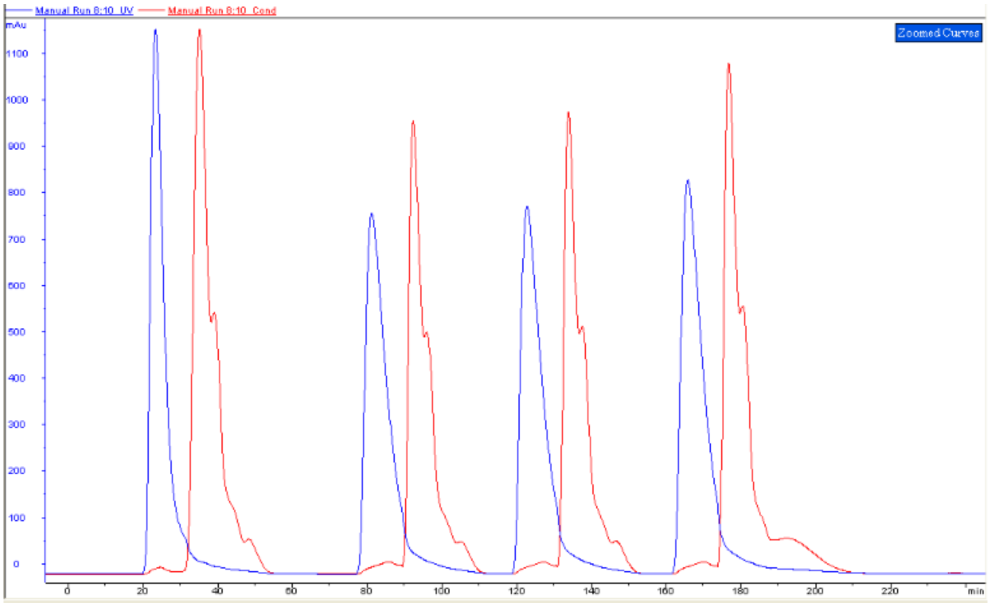

FIG S26 An Äkta chromatogram of preparative size-exclusion chromatographic purification of CDP-3-deoxy-Glc. The blue line corresponds to the UV absorbance of CDP-3-deoxy-Glc at 280 nm and the red line shows the conductivity signal of acetate.

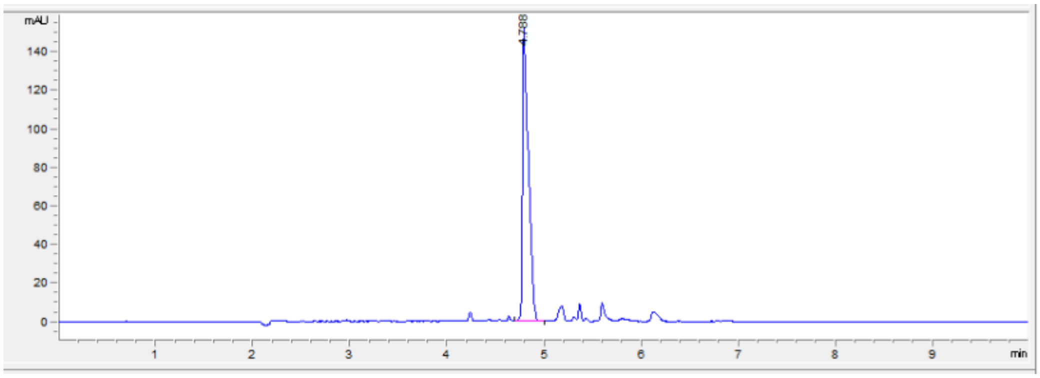

FIG S27 Capillary electrophoresis chromatogram of isolated CDP-3-deoxy-Glc. The desired product was obtained in good purity.

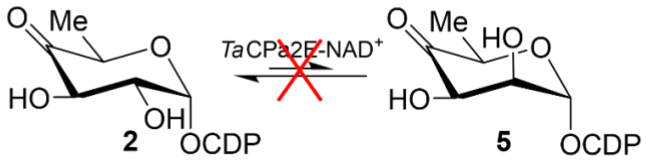

Scheme S5 Schematic representation of the attempted epimerization of **2** (see Fig. 1) to **5** (CDP-6-deoxy-D-*lyxo*-hexopyranos-4-ulose) by *Ta*CPa2E.

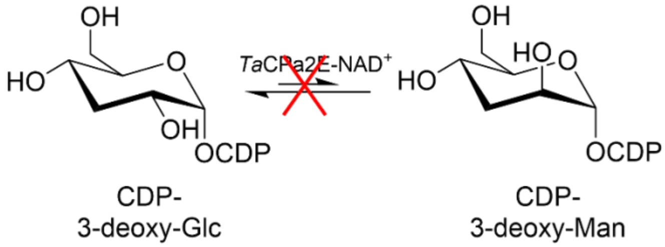

Scheme S6 Reaction scheme of the attempted epimerization of CDP-3-deoxy-Glc to CDP-3-deoxy-Man by *Ta*CPa2E.

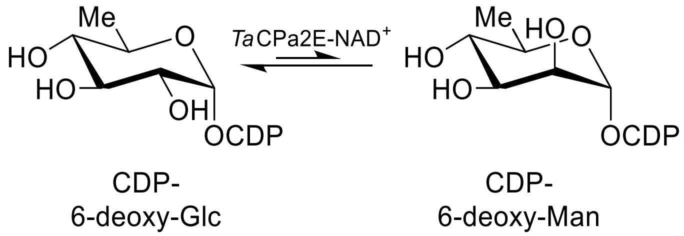

Scheme S7 Reaction scheme of the epimerization of CDP-6-deoxy-Glc to CDP-6-deoxy-Man by *Ta*CPa2E.

Experimental procedures (addition to the main text)

Gene expression and protein purification

Standard conditions for gene expression

Unless stated otherwise, precultures of *E. coli BL21 (DE3)* cells were grown overnight at 37 °C and 100 rpm in 250 mL baffled shake flasks containing 50 mL soy peptone LB medium supplemented with ampicillin (100 µg/mL). Main cultures were prepared similarly in 1000 mL baffled shake flasks and 200 mL gowth medium. Cultures were shaken at 100 rpm and 37 °C in a Certomat® BS-1 incubator (Sartorius) until reaching an optical density of 0.8 – 1. Gene expression was induced by 100 µM isopropyl-β-D-thiogalactopyranoside (IPTG) for 20 h at 18 °C. TB medium was used alternatively for expressing E1 and E3.

Cells were harvested by centrifugation (Sorvall RC-5B) at 4 °C, 2800 g, resuspended in 40 mL binding/loading buffer and disrupted by sonication (2 sec pulse on, 5 sec pulse off, 69% amplitude, 6 min). The lysate was centrifuged (16100 g) at 4 °C for 30 min and the supernatant collected and filtered (0.45 µm) prior to loading onto the pre-equilibrated (loading/binding buffer) columns. Purification was performed on an ÄktaPrime plus system (GE Healthcare, Germany). Proteins were concentrated and re-buffered with 100 mM MOPS (pH 7.5) using Vivaspin filter tubes (10-30 kDa cut-off) followed by addition of 15% glycerol. Aliquots were stored at -20 °C and -80 °C. A Nanodrop spectrophotometer was used to determine protein concentrations at 280 nm absorbance. Purity and molecular size were shown by SDS-PAGE.

Standard conditions for affinity-tag protein purification

His-tagged enzymes were purified using a HisTrap^{TM} HP column (5 ml Ni Sepharose resin, GE Healthcare Life Sciences), HisTag binding buffer (10 mM HEPES, 500 mM NaCl, 5% (w/w)

glycerol, 5 mM imidazole, pH 7.5) and HisTag elution buffer (10 mM HEPES, 500 mM NaCl, 5% (w/w) glycerol, 300 mM imidazole, pH 7.5). Proteins were eluted applying a gradient (100% HisTag elution buffer after 60 min) and a flow rate of 2 mL/min.

Strep-tagged enzymes were purified utilizing a StrepTrap™ HP column (5 ml resin, GE Healthcare Life Sciences), Strep-tag loading buffer (100 mM Tris, 150 mM NaCl, pH 8) and Strep-tag elution buffer (100 mM Tris, 150 mM NaCl, 2.5 mM D-desthiobiotin, pH 8).

Expression and purification of enzymes for general sugar nucleotide synthesis

Inorganic pyrophosphatase (iPPase; from *E. Coli*; UniProt entry: P0A7A9; EC 3.6.1.1; (3)) was expressed in *E. coli* BL21 (DE3) harboring a pET-STREP3_iPPase vector. Galactokinase (GalKSpe4; from *Streptococcus pneumoniae;* UniProt entry: Q97NZ6; EC 2.7.1.6; (4)) was produced in *E. coli* BL21 (DE3) LEMO21 cells carrying a pET28a_GalKSpe4 vector. GalKSpe4 and iPPase were purified by affinity chromatography using Strep-tag. The activity of iPPase was determined as described in the literature (5) and the activity of GalKSpe4 on 3-deoxy-Glc was discovered in the course of an anomeric phosphorylation screening of the respective compound. UDP-glucose pyrophosphorylase (UGPase; from *Bifidobacterium longum*; UniProt entry: B3DRZ1; EC 2.7.7.9; (6)) was produced in *E. coli* BL21 (DE3) Gold cells carrying a pET30_UGPase vector and purified by His-tag affinity chromatography. Activity measurements were performed as described elsewhere (7). Sucrose synthase (AcSuSy; from *Acidithiobacillus caldus;* UniProt entry: A0A059ZV61; EC 2.4.1.13; (8)) was constitutively expressed (8 h) in *E. coli* BL21 (DE3) harboring a pCXP34h_AcSuSy vector. N-acetylhexosamine 1-kinase (NahK; from *Bifidobacterium longum,* UniProt entry: E8MF12; EC 2.7.1.162; (9)) was expressed in *E. coli* BL21 (DE3) carrying a pET21a expression vector. NahK and AcSuSy were purified

utilizing His-tag affinity chromatography. The activities of NahK and AcSuSy were tested as described in the literature (10), (11).

CDP-α-D-paratose synthesis (attempted)

The synthesis route is presented in Fig. S1. Dehydration of 1-20 mM CDP-Glc to **2** (see Fig. 1, compound **2**) was carried out as described before and the intermediate product used for follow up reactions.

Synthesis under oxic conditions

All experiments were performed with *Y. pseudotuberculosis (yp)* or *S. typhi (st)* E1 and E3. The reaction mixture (0.5-10 mL) contained E1 and E3 in various ratios (1/100-100/1) and concentrations starting from 0.01-80 mg/mL E1 and 0.01-20 mg/mL E3. Various NADH concentrations were tested; either equimolar, higher or lower than the concentration of **2** (see Fig. 1). NADH was added either all in once or in fed-batch mode. The buffer used was 100 mM MOPS at pH 7.5. Reactions were carried out at 30-37 °C. As no reaction progress could be monitored, reaction additives comprised pyridoxamine 5´-phosphate (PMP), tris(2-carboxyethyl)phosphine (TCEP), dithiothreitol (DTT), ammonium iron(II)sulfate and iron(III)chloride. Addition of 0.01-10 mg/mL CDP-paratose synthase only led to formation of CDP-6-deoxy-Glc, independent on the amount of NADH or NADPH present. Similar strategies were followed using cell-free extracts of freshly cultivated E1 and E3 instead of isolated enzymes.

Another approach to improve the product formation involved immobilization of his-tagged ypE3 onto hydrophilic, semi-hydrophilic and hydrophobic resins with Fe(III) coating (EnginZyme) to facilitate the interaction of non-tagged ypE1 with immobilized ypE3 to form an active enzyme complex.

Resins (30 mg) were equilibrated in 500 µL buffer (5 mM imidazole, 10 mM HEPES, 300 mM NaCl, 5% v/v glycerol). The resins were mixed for 30 min at 30 rpm on an end-over-end rotator and centrifuged for 10 min (6010 g, 4 °C). The supernatant was removed and 500 µL of fresh cell-free extract containing his-tagged ypE3 was combined with the resins. The aforementioned procedure was repeated followed by the replacement of his-tagged ypE3 by non-tagged ypE1 cell-free extract. The binding procedure was performed three times. Coated E1/E3-beads were added in various concentrations to mixtures containing 1 mM of **2** (see Fig. 1), 1.5 mM NADH and 10 µM PMP in MOPS buffer (100 mM, pH 7.5). Reactions were carried out at 30 °C.

Synthesis under anoxic conditions

A combination of glucose oxidase and catalase was used for oxygen depletion monitored with an OXROB10 oxygen probe (PyroScience, Aachen, Germany). The reaction mixture (4 mL) contained 0.14 mg/mL stE3, 1.1 mg/mL stE1, 2 mg/mL glucose oxidase, 2 mg/mL catalase, 11 mM glucose, 0.3 mM NADH, 0.3 mM of **2** (see Fig. 1) and 0.8 µM PMP in MOPS buffer (100 mM, pH 7.5). The reaction was incubated at 30 °C for 16 h.

In a further attempt, formate dehydrogenase was used in addition to glucose oxidase and catalase to establish a NADH-recycling system. The reaction mixture (4 mL) contained 0.5 mg/mL ypE3, 2 mg/mL ypE1, 2 mg/mL FDH (16 U), 2 mg/mL glucose oxidase, 2 mg/mL catalase, 0.2 mM NAD^+, 2 mM of **2** (see Fig. 1), 0.1 mM PMP in ammonium formate buffer (20 mM, pH 7.5).

CDP-α-D-tyvelose synthesis (attempted)

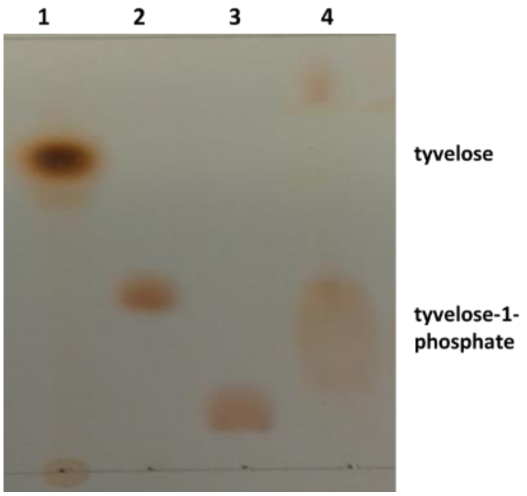

FIG S28 Silica plate of thin-layer chromatographic monitoring of tyvlose-1-phophate synthesis. Chemical phosphorylation of tyvelose was carried out according to published protocols (12), (13). Lane 1: tyvelose (standard), lane 2: glucose (standard), lane 3: glucose-1-phosphate (standard), lane 4: reaction mixture after 16 h showing almost full substrate depletion and tyvelose-1-phosphate formation.

GDP-α-D-glucose synthesis

The synthesis and isolation of the nucleotide-activated glucose GDP-α-D-glucose (GDP-Glc) was performed as described in (14), (15). Modifications encompassed incubation of the precipitated product with 20 g/L baker's yeast for sucrose removal. The desired product was obtained in 72.5 mg yield (> 99% conversion to GDP-Glc) and good purity.

Sugar nucleotide isolation

Anion exchange chromatography

Desired sugar nucleotides were separated from other components by anion exchange chromatography. Enzymes were removed with VivaSpin concentrators (10.000 MWCO, 3220 g) prior to chromatographic purification. Unless stated otherwise, an ÄKTA FPLC system (GE Healthcare) equipped with a XK16/40 column (length 40 cm, i.d. 2.6 cm, column volume 130 mL, GE Healthcare Life Sciences, Germany) and a 10 mL loop were used (Methods). Solutions containing 20 mM and 1 M sodium acetate (pH 4.3) were prepared to function as binding and elution buffers, respectively. Bound compounds were eluted with a linear or a step-wise gradient (see captions in the Supplementary Information for details). Fractions showing UV-absorption at 254 nm were collected and subjected to HPLC analysis. Product containing fractions were pooled and concentrated under reduced pressure to a volume of 20-30 mL prior to gel filtration.

Size-exclusion chromatography

Size-exclusion chromatography was used for sodium acetate removal from concentrated product solutions. The Superdex G-10 column (see Methods) was connected to an ÄKTA FPLC system and a 5 mL sample loop. Concentrated samples from anion exchange chromatography were applied directly. Deionized water at a flow rate of 3 mL/min was used for elution and sugar nucleotides were detected by UV-absorption at 254 nm. Product-fractions were pooled and concentrated under reduced pressure (final volume ~10 mL) prior to lyophilization.

References

1. Tanaka H, Yoshimura Y, Hindsgaul O. 2013. A simple chemical synthesis of sugar nucleoside diphosphates in water. *Curr Protoc Nucleic Acid Chem* **13.12**:1–10.
2. Smith P, Lin A, Szu PH, Liu HW, Tsai SC. 2006. Biosynthesis of a 3,6-dideoxyhexose: Crystallization and X-ray diffraction of CDP-6-deoxy-L-threo-D-glycero-4-hexulose-3-dehydrase (E1) for ascarylose biosynthesis. *Acta Crystallogr Sect F Struct Biol Cryst Commun* **62**:231–234.
3. Lahti R, Pitkäranta T, Valve E, Ilta I, Kukko-Kalske E, Heinonen J. 1988. Cloning and characterization of the gene encoding inorganic pyrophosphatase of Escherichia coli K-12. *J Bacteriol* **170**:5901–5907.
4. Chen M, Chen LL, Zou Y, Xue M, Liang M, Jin L, Guan WY, Shen J, Wang W, Wang L, Liu J, Wang PG. 2011. Wide sugar substrate specificity of galactokinase from Streptococcus pneumoniae TIGR4. *Carbohydr Res* **346**:2421–2425.
5. Pfeiffer M, Bulfon D, Weber H, Nidetzky B. 2016. A Kinase-Independent One-Pot Multienzyme Cascade for an Expedient Synthesis of Guanosine 5′-Diphospho-d-mannose. *Adv Synth Catal* **358**:3809–3816.
6. Kotake T, Yamaguchi D, Ohzono H, Hojo S, Kaneko S, Ishida HK, Tsumuraya Y. 2004. UDP-sugar pyrophosphorylase with broad substrate specificity toward various monosaccharide 1-phosphates from pea sprouts. *J Biol Chem* **279**:45728–45736.
7. Borg A, Dennig A, Weber H, Nidetzky B. 2020. Mechanistic characterization of UDP-glucuronic acid 4-epimerase. *FEBS J*.
8. Diricks M, De Bruyn F, Van Daele P, Walmagh M, Desmet T. 2015. Identification of sucrose synthase in nonphotosynthetic bacteria and characterization of the recombinant enzymes. *Appl Microbiol Biotechnol* **99**:8465–8474.

9. Nishimoto M, Kitaoka M. 2007. Identification of N-acetylhexosamine 1-kinase in the complete lacto-N-biose I/galacto-N-biose metabolic pathway in Bifidobacterium longum. *Appl Environ Microbiol* **73**:6444–6449.

10. Li Y, Yu H, Chen Y, Lau K, Cai L, Cao H, Tiwari VK, Qu J, Thon V, Wang PG, Chen X. 2011. Substrate promiscuity of n-acetylhexosamine 1-kinases. *Molecules* **16**:6396–6407.

11. Diricks M, Gutmann A, Debacker S, Dewitte G, Nidetzky B, Desmet T. 2017. Sequence determinants of nucleotide binding in Sucrose Synthase: Improving the affinity of a bacterial Sucrose Synthase for UDP by introducing plant residues. *Protein Eng Des Sel* **30**:143–150.

12. Edgar LJG, Dasgupta S, Nitz M. 2012. Protecting-group-free synthesis of glycosyl 1-phosphates. *Org Lett* **14**:4226–4229.

13. Gudmundsdottir A V., Nitz M. 2008. Protecting group free glycosidations using p-toluenesulfonohydrazide donors. *Org Lett* **10**:3461–3463.

14. Gutmann A, Nidetzky B. 2016. Unlocking the Potential of Leloir Glycosyltransferases for Applied Biocatalysis: Efficient Synthesis of Uridine 5′-Diphosphate-Glucose by Sucrose Synthase. *Adv Synth Catal* **358**:3600–3609.

15. Lemmerer M, Schmölzer K, Gutmann A, Nidetzky B. 2016. Downstream Processing of Nucleoside-Diphospho-Sugars from Sucrose Synthase Reaction Mixtures at Decreased Solvent Consumption. *Adv Synth Catal* **358**:3113–3122.
